# Phytotherapeutic options for the treatment of epilepsy: pharmacology, targets, and mechanism of action

**DOI:** 10.3389/fphar.2024.1403232

**Published:** 2024-05-24

**Authors:** Abdul Waris, Ata Ullah, Muhammad Asim, Rafi Ullah, Md. Rafe Rajdoula, Stephen Temitayo Bello, Fahad A. Alhumaydhi

**Affiliations:** ^1^ Department of Biomedical Sciences, City University of Hong Kong, Kowloon, Hong Kong SAR, China; ^2^ Department of Neurosciences, City University of Hong Kong, Kowloon, Hong Kong SAR, China; ^3^ Centre for Regenerative Medicine and Health (CRMH), Hong Kong, Hong Kong SAR, China; ^4^ Department of Botany, Bacha Khan University Charsadda, Charsadda, Pakistan; ^5^ Department of Medical Laboratories, College of Applied Medical Sciences, Qassim University, Buraydah, Saudi Arabia

**Keywords:** epilepsy, plant extracts, phytochemicals, antiepileptic, anticonvulsive, antagonist, agonist

## Abstract

Epilepsy is one of the most common, severe, chronic, potentially life-shortening neurological disorders, characterized by a persisting predisposition to generate seizures. It affects more than 60 million individuals globally, which is one of the major burdens in seizure-related mortality, comorbidities, disabilities, and cost. Different treatment options have been used for the management of epilepsy. More than 30 drugs have been approved by the US FDA against epilepsy. However, one-quarter of epileptic individuals still show resistance to the current medications. About 90% of individuals in low and middle-income countries do not have access to the current medication. In these countries, plant extracts have been used to treat various diseases, including epilepsy. These medicinal plants have high therapeutic value and contain valuable phytochemicals with diverse biomedical applications. Epilepsy is a multifactorial disease, and therefore, multitarget approaches such as plant extracts or extracted phytochemicals are needed, which can target multiple pathways. Numerous plant extracts and phytochemicals have been shown to treat epilepsy in various animal models by targeting various receptors, enzymes, and metabolic pathways. These extracts and phytochemicals could be used for the treatment of epilepsy in humans in the future; however, further research is needed to study the exact mechanism of action, toxicity, and dosage to reduce their side effects. In this narrative review, we comprehensively summarized the extracts of various plant species and purified phytochemicals isolated from plants, their targets and mechanism of action, and dosage used in various animal models against epilepsy.

## 1 Introduction

**FIGURE 1:**
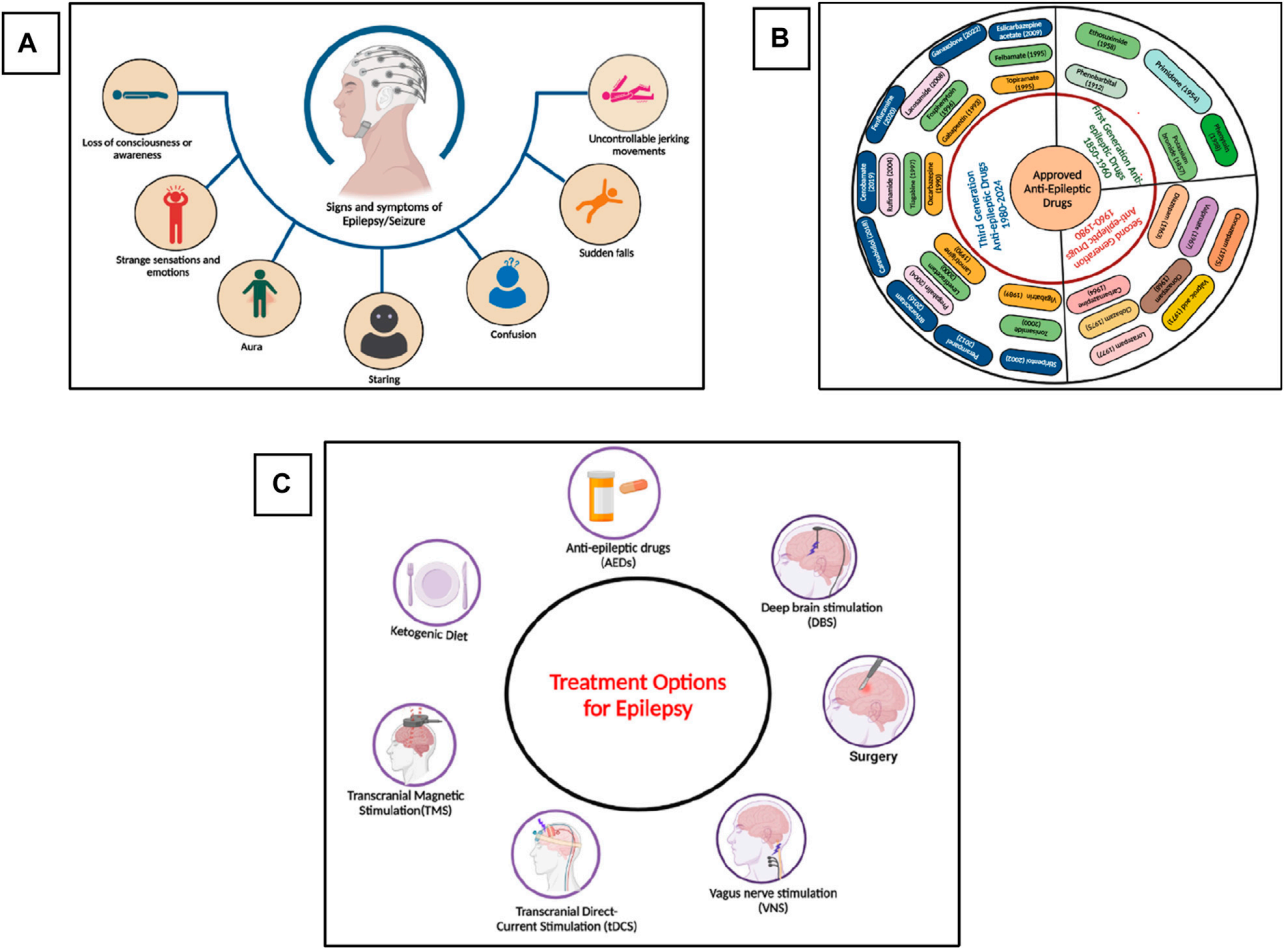
**(A)** Signs and symptoms of seizure and epilepsy, **(B)** Approved and Marketed AEDs by USFDA, **(C)** Different treatment options for the treatment of epilepsy. (Figures were generated using Biorender online version).

Epilepsy is one of the most common chronic and heterogeneous neurological disorders, affecting more than 60 million individuals worldwide (Moshé et al., 2015). Epilepsy is characterized by seizures, which are abnormal electrical activities in the brain resulting in motor, sensory, or psychomotor experiences (Potnis et al., 2020). These activities in the brain occur due to the imbalance of excitatory and inhibitory neuronal pathways, and the tendency to develop seizures again and again leads to epilepsy. According to the latest report of the International League Against Epilepsy (ILAE), *epilepsy* is defined as” two or more unprovoked seizures greater than 24 h apart, or single unprovoked seizure in an individual with 60% chances of having another seizure in the next 10 years, or an epilepsy syndrome (clinical, EEG, clinical, genetics and age-dependent features) (Falco-Walter, 2020; Specchio et al., 2022; Wirrell et al., 2022). The signs and symptoms of epilepsy are shown in Figure 1A. The global prevalence of epilepsy is 1%, and among them, 80% of inhabitants live in low-income and lower-middle-income countries. According to the World Health Organization (WHO), about 75% of the affected individuals do not get proper treatment for the management of epilepsy, and this percentage has also reached 90% in some low-income countries, as these anti-epileptic drugs (AEDs) are inaccessible, too expensive, or have unwanted side effects (O’Donohoe et al., 2020; Pironi et al., 2022). Currently, more than thirty pharmacological drugs have been approved by the United States Food and Drug Administration (USFDA) and are available in the market (Perucca, 2021), as shown in Figure 1B. Almost 70% of the affected people become seizure-free after proper medication with the available AEDs. Still, 25%–35% of people show resistance to the current medications and are termed refractory, intractable, or drug-resistant epilepsy, and almost 50% of the sudden deaths from epilepsy belong to the drug-resistant epilepsy group (Mula, 2021; Borowicz-Reutt et al., 2023; Rocha et al., 2023). Currently, different treatment options are available, as shown in Figure 1C, including pharmacological treatment, but every treatment strategy has its own limiting factors. Due to the different limitations of the treatment options against epilepsy, phytotherapeutic treatment or herbal medicines got huge attention worldwide due to its safety, low toxicity, easy availability, cost-effectiveness, and multitargeting ability to focus on the various medicinal plants and their phytochemicals for the treatment of epilepsy.

Medicinal plants are the main sources of pharmaceuticals in early discovery. Even in the 21st century, 80% of the population in lower-income countries depends on extracts of various plants for the preventive and curative treatment of various diseases, including neurological disorders, malaria, mycetoma, etc. Plant species such as *Bacopa monnieri, Centella Asiatica, Curcuma longa, Cyperus rotundus, Morinda citrifolia, and Withania somnifera are among the most common plants used for treating neurological disorders* (Berdigaliyev and Aljofan, 2020; Bhat and Sharma, 2020; Fitzgerald et al., 2020; Porras et al., 2020). Medicinal plants generate different types of substances that have promising therapeutic potential, which are termed phytochemicals or bioactive compounds. These phytochemicals have the potential to target specific receptors in various diseases and perform therapeutic activities. The WHO also recommends the use of plant-derived medicines with better safety and therapeutic efficacy in various countries (Palhares et al., 2015). In the last few years, scientists have focused on natural products, especially medicinal plants, to find cost-effective, safer, and potent agents for preventive, prophylactic, and other medicinal purposes. Many studies have been carried out to evaluate the therapeutic potential of various types of phytochemicals against epilepsy at a pre-clinal stage. (Kaur et al., 2021; He et al., 2023). It has been reported that numerous phytochemicals isolated from plants have promising potential to reach various drug targets in epileptic animal models (Chipiti et al., 2021; Subedi and Gaire, 2021; Zadali et al., 2022; Carreño-González et al., 2023). These phytochemicals can interact with different types of receptors in the central nervous system (CNS), such as N-methyl-D-aspartate receptor (NMDAR), Gamma-aminobutyric acid receptor (GABAAR), and α-amino-3-hydroxy-5-methyl-4-isoxazolepropionic acid receptor (AMPAR), channels like voltage gated sodium, potassium, and calcium channels, and other enzymes and pathways that play a significant role in the initiation and propagation of epileptic seizures. In this narrative review, we summarized the up-to-date information on various plants/medicinal plant extracts, specific phytochemicals isolated from various plants, their mechanism of action, and how these interact with various targets and help in the control of seizures and treatment of epilepsy.

## 2 Approved drugs and pharmacological targets

### 2.1 Current treatment options

Epilepsy affects people of all ages, with a high prevalence in people aged more than 65. Overall, 25%–35% of people still show resistance to the available AEDs. (Campos-Bedolla et al., 2022; Asadi-Pooya et al., 2023). To overcome this issue, different treatment options have been developed, such as surgery (if the seizure is focal, i.e., originated from a specific region of a brain such as in the case of temporal lobe epilepsy in which the seizure originated from the hippocampus), ketogenic diet, deep brain stimulation (DBS), vagus nerve stimulation (VNS), transcranial magnetic stimulation (TMS), and transcranial direct current stimulation (tDCR) (Harris and Angus-Leppan, 2020; Löscher et al., 2020; Asadi-Pooya et al., 2023). Each of these approaches has its own limitations, such as availability, cost, success rate, etc (Thijs et al., 2019). Currently, the most common way to treat epilepsy is pharmacological drugs, as it is easily available and used, have a high success rate, and are cost-effective as compared to other treatment approaches. In the 19th century, potassium bromide and some medicinal plants were used to treat seizures and epilepsy. Then, at the start of the 20th century, phenobarbital, which is a GABAA receptor agonist, was officially approved for the treatment of epilepsy in 1912. From 1850 to 1960, mostly five drugs were commonly used to treat seizures and epilepsy (Perucca, 2021; Tomson et al., 2023), as shown in Figure 1B. Then, until 1980, eight more drugs were approved by the USFDA for the treatment of epilepsy. Later in the 21st century, the advancement in the field of neurosciences, especially in the mechanism of disease and diagnostic approaches, the development of drugs against various neurological disorders, especially epilepsy, got much attention, and within the last 3 decades, more than twenty drugs have been approved by the USFDA for the treatment of epilepsy (Perucca, 2021). AEDs that have been approved by the US FDA from 1950 till 2024 are summarized in Figure 1B. Researchers are now focusing on the drugs’ cost-effectiveness and high success rate, and several drugs and other treatment approaches are currently in various phases of clinical trials (ClinicalTrials.gov 2024).

### 2.2 Pharmacological targets of the approved AEDs

Epilepsy develops due to the imbalance of action or resting membrane potential (Scharfman, 2007). Various receptors, membrane channels, and enzymes have been involved in the imbalance of depolarization and hyperpolarization of neurons, which then leads to a seizure (Armijo et al., 2005). Some of these are present on the presynaptic neuron, while some are on postsynaptic neurons. These protein or membrane channels include voltage-gated sodium channels responsible for the sodium ion (Na^+^) influx in the cell during normal functioning (Mantegazza et al., 2010). The sodium channels are normally found in three states (see Section 7); in case of seizure, these channels are in an active state for a longer time, or it takes more time to go to an inactive state due to mutation occurring in the proteins subunits of the channels or due to any other brain insult (Crill, 1996; Mantegazza et al., 2010). So when more sodium channels are active for a longer time, a high influx of Na^+^ will occur in presynaptic neurons, leading to the imbalance of normal signalling in the neuron and generating an imbalance in the excitatory and inhibitory system of the neurons, leading to a seizure. (Kaplan et al., 2016). Some approved drugs such as Rufinamide, Oxcarbazepine, and Phenytoin targeting Na^+^ channels to control the seizures and the treatment of epilepsy (Perucca, 2021).

At the end of presynaptic neurons, calcium channels are present, which allow the calcium ion (Ca^2+^) to go inside the cell and perform normal function, which helps in the release of the neurotransmitter (Meir et al., 1999). However, in the case of epilepsy, dysfunction of these important channels occurs due to mutations in the subunits or any other brain insult. Therefore, the concentration of Ca^2+^ in the cell is out of control, and hence, an influx of more Ca^2+^ occurs, which leads to the release of more neurotransmitters and disturbs the normal function of the cell (Carvill, 2019). Several clinically approved drugs such as Gabapentin, Pregabalin, and Ethosuximide block these channels and hence control the seizure and epilepsy (Tomson et al., 2023).

Similarly, during normal cell functioning, Na^+^ influx and potassium (K^+^) outflux occur to maintain normal cell function. However, during the seizure, the voltage-gated potassium channels (Kv) fail to efflux enough K^+^ ions out of the cell to maintain a normal level of ions inside the cell, which causes an imbalance of the action potential and again leads to the disturbance of excitatory and inhibitory cycle of the neurons and finally develops seizure (Gao et al., 2022). In addition, a few more channels and enzymes are involved in the initiation and propagation of seizure (Rho and Boison, 2022; Perucca et al., 2023a; Perucca et al., 2023b).

**FIGURE 2:**
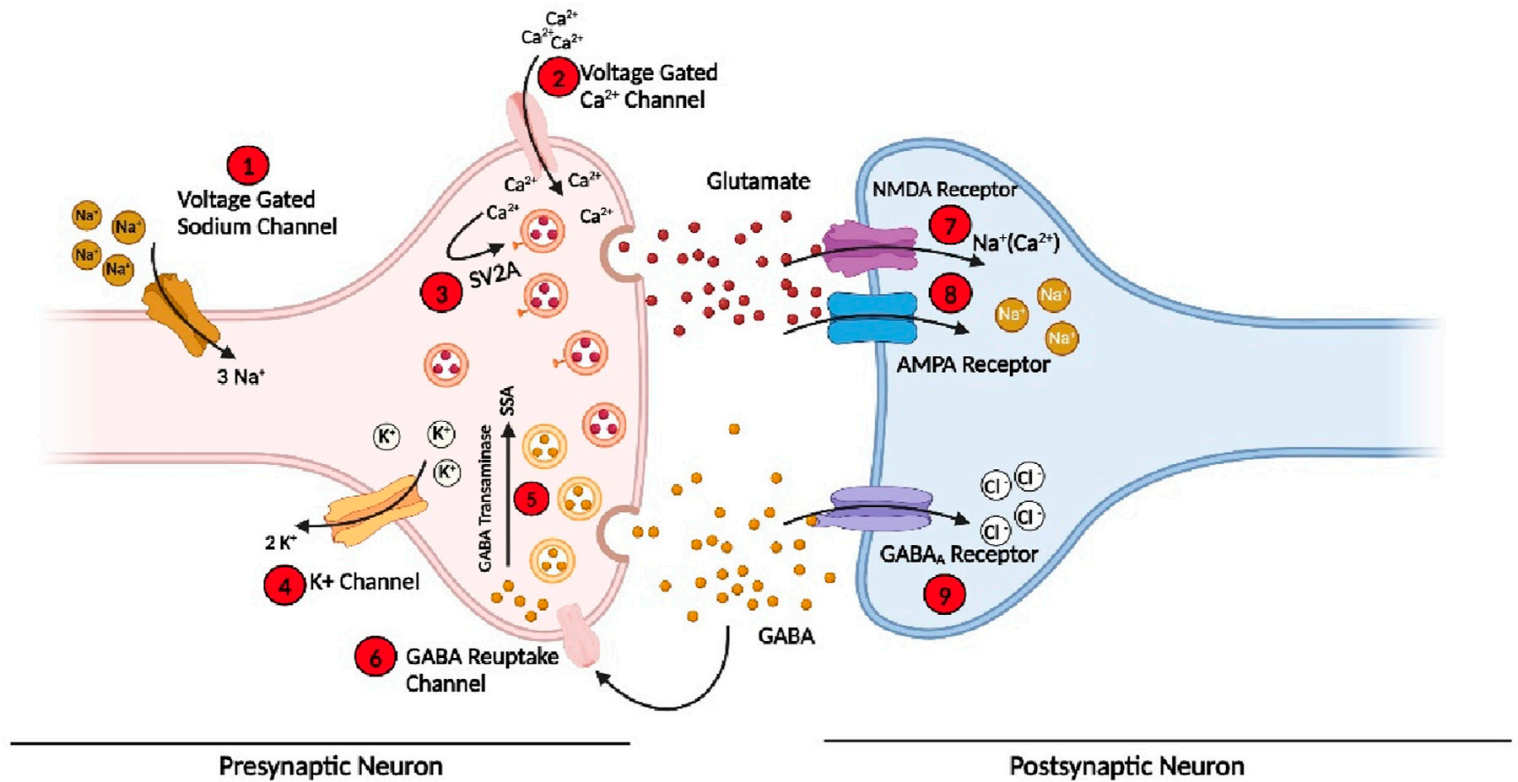
Various receptors, membrane protein channels, and enzymes involved in the initiation and propagation of seizure and the targets for the current AEDs and future drugs. (Bio-render online version was used for this figure; for abbreviations, please see the list of abbreviations).

Similarly, on postsynaptic neurons, three main important receptors are present, which play a pivotal role in the cell signalling and development of epilepsy and are also the main target of the current and development of new AEDs. These receptors or channels include NMDARs and AMPARs to which glutamate, a primary excitatory neurotransmitter attached, opens the channels and allows more positive ions (Na^+^/Ca^2+^) to go inside the postsynaptic neuron and generate an action potential (Sivakumar et al., 2022; Chen et al., 2023). Some clinically approved AEDs, such as Perampanel and Valproate, target these two receptors on the postsynaptic neuron to control the generation or propagation of the action potential in epilepsy (Tomson et al., 2023). In addition, GABARs, to which GABA, an inhibitory neurotransmitter, binds, are the most important target to control seizures and treat epilepsy; they are also present on the postsynaptic neuron, which allows chlorine ions (Cl-) to go inside the cell to maintain and balance the internal environment (Fu et al., 2022; Akyuz et al., 2023; Bryson et al., 2023). Among the currently approved AEDs, more than 35% of the drug target is GABAR, which acts as an agonist to open the channels for a longer time. These includes Stiripentol, Vigabatrin, Diazepam, and Clonazepam (Perucca, 2021). Similarly, some drugs target various enzymes involved in the development of seizures and epilepsy. The details of channels, receptors, and enzymes involved in the development of seizures and epilepsy, as well as the main target of the drugs, are shown in Figure 2.

## 3 Plants/medicinal plants and phytochemicals

Phytochemicals, also termed phytonutrients, are bioactive compounds produced by different plants in various parts such as stem, leaves, roots, and flowers as a result of primary and secondary metabolism (Nahar et al., 2020). These bioactive compounds play a significant role in the growth of plants or a host’s defense against various pathogens and competitors (Bose et al., 2020). There are countless phytochemicals, however, only a small number have been identified (Xiao and Bai, 2019). Phytochemicals include polyphenols, alkaloids, carotenoids, terpenoids, flavonoids, limonoids, coumarins, indoles, isoflavones, lignans, organosulfur, catechins, furyl compounds, phenolic acids, stilbenoids, isothiocyanates, saponins, procyanidins, phenylpropanoids, anthraquinones, ginsenosides and so on (Xiao and Bai, 2019; Welcome, 2020). These phytochemicals have been considered one of the most widely used treatment options in different countries for the treatment of various diseases such as cancer, neurological disorders, and other virological infections (Behl et al., 2021; George et al., 2021; Yadav, 2021; Lim and Park, 2023). These phytochemicals have multifunctional properties such as antioxidant, anti-inflammatory, antidiabetic, antimicrobial, anticancer, and immunomodulatory, and they act as antagonists and agonists for various types of inhibitory and excitatory receptors in the body, especially in the CNS. Blood brain barrier (BBB) is one of the main hurdles in developing drugs against various neurological disorders, as most of the compounds with high molecular weight cannot cross and attenuate BBB (Akhtar et al., 2021; Chowdhury et al., 2021; Xiong et al., 2021; Yadav, 2021; Correia et al., 2022). However, it has been reported that most phytochemicals such as allopregnanolone, asiaticoside, berberine, catalpol, curcumin, and many more can cross BBB and attenuate the inflammation and hyperpermeability in various pathological conditions (Yadav, 2021).

Several studies have been reported on the efficacy of plant extracts and phytochemicals against various diseases related to the central nervous system (Ayeni et al., 2022; Luthra and Roy, 2022). Curcumin, generally used as a spice in food, is reported to have a promising anti neurodegenerative effect in animal models and also in humans, especially against dementia. In addition, spicatoside, resveratrol, broccoli, and many more have been studied for their effect against various neurological and neuroprotective efficacy (Subedi et al., 2020). Moreover, medicinal plants (phytochemicals and metabolites) have been extensively used for the treatment of various neurological diseases, especially epilepsy in most of the low- or middle-income countries (Asiimwe et al., 2021; Birhan, 2022). These plants, as a treatment option for epilepsy, have very low cost, high availability, and fewer side effects as compared to other treatment options (Sucher and Carles, 2015; Kaur et al., 2021). These play a significant role in the treatment of epilepsy by interfering with the excitatory pathway or inhibitory pathway. Hundreds of plants have been extensively studied *in vitro* and *in vivo* (Khan et al., 2020; Kaur et al., 2021). However, only a few have reached clinical trials. Similarly, Cannabidiol is a bioactive metabolite found in marijuana, a plant species that has high antiepileptic properties (Kaur et al., 2021). Therefore, in 2018, the USFDA approved CBD-rich Epidiolex against two severe forms of epilepsy in children (Golub and Reddy, 2021). In addition, CBD-based treatment is currently in phase 4 clinical trials for the treatment of refractory epilepsy (Kaur et al., 2021). A lot of work has been done on the use of plant extract and isolated phytochemicals against epilepsy; we will discuss their targets and the mechanism for the treatment of epilepsy.

## 4 Modulation of the inhibitory system

The imbalance in the excitatory and inhibitory neuronal systems in the brain leads to seizures and epilepsy. Different types of neurotransmitters in the brain play an important role in the pathophysiology of epilepsy. Among them, GABA and glutamate are the key neurotransmitters directly involved in the excitatory and inhibitory pathways (Jangra and Budhwar, 2022). Among inhibitory neurons and neurotransmitters, GABA is the key inhibitory neurotransmitter released in the brain synapses, accounting for more than 40% (Treiman, 2001). Glutamic acid decarboxylase (GAD) enzyme converts glutamate to GABA, which is then released from the presynaptic GABAergic neuron and acts on the post-synaptic neuron. GABA has three receptors, i.e., GABAA, GABAB, and GABAC, on the post-synaptic neurons (Briggs and Galanopoulou, 2011), and among them, GABAA is of critical importance as this receptor is involved in controlling and balancing action potential and main target of the AEDs. When GABA is releases from the presynaptic neuron, it attaches to the GABAAR on the post-synaptic neuron, allowing the influx of Cl^−^ ion and hence controlling the action potential by balancing the internal environment (Kullmann et al., 2005). In addition, there is a GABA reuptake channel on the presynaptic neuron, which allows the influx of GABA after performing function or extra GABA released in the synapses, as shown in Figure 2. In the case of epilepsy, dysregulation occurs in the inhibitory system, especially in GABAAR, lower production of GABA by GABAergic neurons, and/or catabolism of GABA, and hence, imbalance occurs, which leads to epilepsy (He et al., 2023). These inhibitory neurotransmitters and receptors are the main target of the drugs, and about 35% of the available pharmacological drugs target the inhibitory pathway of CNS.

It has been reported that several plant extracts and isolated phytochemicals from various plants have the ability to modulate the inhibitory system in several ways. (Kennedy and Wightman, 2011). One of the possible mechanisms reported is plant extracts, and their phytochemicals could increase the levels of GABA in the brain by activating enzymes responsible for the synthesis of GABA. Recently, Ali Mohammad and coworkers evaluated the epileptic potential of the stem bark extract of the medicinal plant *Vateria indica Linn*, which is commonly known as White Damamr. The authors first analyzed the phytochemical profile of the plant and found that the plant is rich in various bioactive compounds, especially flavonoids, glycosides, phenolics, tannins, saponins, polysaccharides, and steroids. They used three different mice models, the maximal electrical shock (MES) model of epilepsy, ionized (INH) induced model, and pentylenetetrazol (PTZ) model, and evaluated the effect of the stem bark extract against various parameters such as seizure onset time, duration of convulsions, as well as estimated the level of GABA in the brain using two different doses of 250 and 500 mg/kg and compared with the standard AEDs diazepam. The authors concluded that the plant extract showed strong antiepileptic activities by increasing the onset time of the seizure and decreasing the duration of convulsions. The authors documented that the extracts lead to an increase in the level of GABA in the brain, especially in INH and PTZ models, which could be the possible mechanism of the extract (Alshabi et al., 2022). The authors did not mention the mechanism of how the extract, or its phytochemicals, increases the GABA level in the brain. However, as previously reported, some phytochemicals increase the activity of L-glutamate decarboxylase (L-GAD), an enzyme responsible for the conversion of L-glutamate to GABA, and therefore, the level of GABA increases in the brain, especially in the hippocampus which leads to the activation of inhibitory pathway and help in the control of seizure (Kandeda et al., 2021a). In addition, there are some author limitations of this study, such as the death rate in the MES group, which was 50%, which is higher and could not be acceptable as no reasons were provided. Similarly, further extensive investigation could be carried out to study the proper mechanism of action of the extracts; safety, cytotoxicity, other side effects, and specific dosage are critical.

Similar results on the effect of plant extract on the alteration of GABA levels have been reported by Kandeda et al. (2021b). They used a hydroethanolic extract of roots of *Pergularia daemia* against a PTZ-induced temporal lobe epileptic mice model. The authors used four different concentrations of 1.6, 4, 8, and 16 mg/kg and compared the results with the clinically approved drugs sodium valproate. They reported that the concentrations 4–16 mg/kg have a promising antiepileptic potential and protected the mice against myoclonic jerk and generalized tonic-clonic jerk compared to the sodium valproate. Similarly, all the doses increased the seizure score. As altered GABA, GABA-T and L-GAD have been reported in PTZ kindling-induced models. Here in this study, the authors revealed that this plant extract increased the concentration of GABA by 240% in groups treated with 4 and 8 mg/kg, which is almost 3-fold higher than the increase in the sodium valproate group (96%). They also confirmed that PTZ leads to the reduction of GABA levels by 71%. In addition, they also documented the effect of this plant’s extract against the inflammatory biomarker, cognitive functions, hippocampal neuronal damage, and oxidative stress marker, and revealed that it leads to the improvement of all these parameters as this plant is rich in bioactive compounds such as cardenolides, phenols, alkaloids, flavonoids, triterpenes, saponins, glycosides, cardiac glycosides, saponins, and carbohydrates (Sridevi et al., 2014; Chandak and Dighe, 2019). Similarly, the roots extract of *P. daemia* has also been investigated against the pilocarpine (PILO)-induced (Kandeda et al., 2017) and kainic acid (KA) induced mice models (Kandeda et al., 2021a); in both studies, it showed promising potential against epilepsy. Therefore, this plant could be used as a treatment option against temporal lobe epilepsy after further investigation of its safety and toxicity. Similar studies including (Yempala et al., 2014; Kandeda et al., 2022) have also been reported on the alteration of GABA level by various isolated phytochemicals or plant extracts. Different types of plant extracts extracted phytochemicals, the model and species used, and the dosage reported against epilepsy targeting GABA have been summarized in Table 1.

**TABLE 1:** Effect of plant extracts and isolated phytochemicals from various plants, their mechanism of action, against various models epilepsy through the modulation of inhibitory system.

S. No	Plant (s)	Phytochemical	Plant extract/Isolated phytochemical used	Model(s) used	Specie (s) used	Dose (s)	Target(s)/Mechanism of action	References
1	*Eriobotrya japonica and Hedyotis diffusa*	Ursolic acid	Phytochemical	PILO induced	Male Sprague–Dawley rats	20 and 100 mg/kg	Attenuated GABAergic interneuron loss	Liu et al. (2022)
2	*Prunus dulcis and Chlorophora tinctoria*	Morin	Phytochemical	PTZ induced	Swiss albino mice	20, 40 mg/kg	Increased GABA levels	Kandhare et al. (2018)
3	*Ginkgo biloba*	Rutin	Phytochemical	KA induced	BALB/c mice	100 and 200 mg/kg	Interacted with GABAAR	Nassiri-Asl et al. (2013)
4	*Canarium sweinfurthii*	Flavonoids, sterols, phenolic compounds, tannins, terpenes, and alkaloids	Stem barks extract	4-AP, PILO and PTZ	Swiss albino mice	11.9 mg/kg	Increased GABA and decreased GABA-T	Kandeda et al. (2021c)
6	*Asparagus racemosus*	Quercetin	Roots extract	Strychnine, MES and PTX	Swiss albino mice	(250 and 500 mg/kg	Modulation of GABAA receptors	Shastry et al. (2015)
7	*Coleus amboinicus*	Alkaloids, flavanoids, tannins, triterpenoids, saponins	Whole plant extract	MES and PTZ induced	Swiss albino mice	100 mg/kg	Increased GABA levels	Bhattacharjee and Majumder, (2013)
8	*Annona senegalensis Pers*	alkaloids, terpinoids and saponins	Roots extract	PILO and PTX induced	Mice	150 mg/kg	Increased GABA levels	Konate et al. (2012)
9	*Tapinanthus globiferus*	Saponins, Tannins, Glycosides, Protein, Steroids and Flavonoids	Whole plant extract	PTZ and MES induced	Mice	250, 500, and 1,000 mg/kg	GABAAR modulation and activation of benzodiazepines	Abubakar et al. (2016)
10	*Coffea canephora*	Ferulic aci	Phytochemical	PTZ induced	Wistar rats	75 and 100 mg/kg	Increased GABAergic neurotransmission	Hassanzadeh et al. (2017)
11	*Globimetula braunii*	Saponins, carbohydrates, flavonoids, tannins, anthraquinones and steroids	Leaves extract	PTZ induced	Swiss albino mice	150 mg/kg	Enhancement of GABAergic neurotransmission	Aliyu et al. (2014)
12	*Bixa orellana*	Alkaloids, tannins, proteins, terpenoids, flavonoids, and steroid	Leaves extract	INH induced	Swiss albino mice	200, 400and 800 mg/kg	Modulation of GABAAR	Offiah et al. (2023)
13	*Swertia corymbosa*	Gentiopicroside and swertianin	Aerial parts extract	PTZ, INH, and MES induced	Swiss albino mice	125–500 mg/kg	Modulation of benzodiazepines receptor of GABAAR	Mahendran et al. (2014)
14	*Amaranthus spinosus*	Alkaloids, saponins, cardiac glycosides, flavonoids, carbohydrates, anthraquinones, tannins, and triterpenes	Leaves extract	PTZ induced	Albino mice	400 and 800 mg/kg	Enhancement of GABA	Usman et al. (2023)
15	*Culcasia falcifolia*	Alkaloids, flavonoids, saponins, tannins, polyphenols, and glycosides	Leaves extract	PTZ induced	Mice	200 and 400 mg/kg	Activating GABAA receptors	Gracelyn Portia et al. (2018)
16	*Pandanus odoratissimus Linn*	Alkaloids, lipids, terpenes, triterpenoids, flavonoids and coumarins	Leaves extract	MES, PTX, and strychnine induced	Swiss Albino mice	100 and 200 mg/kg	Increased GABA-mediated inhibition	Adkar et al. (2014)
17	*Acalypha fruticosa*	Alkaloids, steroids, flavanoids and lipids	Aerial parts extract	PTZ, MES, and INH induced	Swiss albino mice	300 mg/kg	enhanced GABAergic neurotransmission	Govindu and Adikay, (2014)
18	*Achyranthes aspera Linn*	Alkaloids, carbohydrates, glycosides, flavonoids, saponins, phenolic compounds, amino acids and steroids	Roots extracts	PTZ, picrotoxin and bicuculline induced	Swiss Albino mice	5 and 10 mg/kg	Increased GABA levels	Gawande et al. (2017)
19	*Annona senegalensis*	Kaurenoic acid, a diterpenoid	Roots extract	PTZ induced	albino mice	200, 400 and 800 mg/kg	Modulation of GABAAR	Okoye et al. (2013)
20	*Viscum album L*	Alkaloids, glycosides, flavonoids, saponins, sterols, tannins, phenolic compounds, amino acids, proteins, fatty acids, carbohydrates, volatile oils and terpenes	Leaves extract	MES, INH and PTZ induced	Swiss albino mice and Wistar albino rats	50 and 150 mg/kg	Enhancing the GABAergic system	Gupta et al. (2012)
21	*Viola tricolor*	ethyl acetate and n-butanol fractions	Whole plant extract	PTZ and MES induced	Albino mice	100, 200, and 400 mg/kg	Modulation of the GABAA receptor complex	Rahimi et al. (2019)
22	*Trachyspermum ammi (L.)*	Thymol	Seeds extract	Strychnine induced	Rates	50 mg/kg	GABAA receptors	Rajput et al. (2013)
23	*Silybum marianum*	Phenols and flavonoids	Seeds extract	PTZ induced	Albino mice	300 mg/kg	Modulation of GABAAR	Waqar et al. (2016)
24	*Caralluma dalzielii*	Flavonoids, tannins, saponins, carbohydrates, steroids, glycosides, cardiac glycosides and phenols, Hexadecanoic acid methyl ester (palmitic acid)	Aerial parts extract	strychnine, PTZ, MES induced	Ranger cockerel	250, 500 and 1,000 mg/kg	Activation of GABAAR	Ugwah-Oguejiofor et al. (2023)
25	*Ceiba pentandra (L.)*	Tannins, terpenoids, saponins, phytosterols, glycosides, flavonoids and alkaloids	Leaves extract	MES, 4-AP, PTZ), and PIC induced	ICR mice	100 mg/kg	Modulation of GABAergic Pathway	Sarfo et al. (2022)
26	*Newbouldia laevis*	Tannins, flavonoids, alkaloids, triterpenes, saponins, and carbohydrates	Leaves extract	Strychnine, PTZ, and MES induced	Albino mice	400 mg/kg	Increased GABA levels	Ukwubile et al. (2023)
27	*Caladium bicolor aiton*	Carbohydrate, tannins, proteins, alkaloids, flavonoids, steroidal nucleus, cardiac glycosides and phenolic compounds	Leaves extract	Strychnine, PTZ, and MES induced	Swiss albino mice	100 and 200 mg/kg	activation of inhibitory GABAergic receptors	Akhigbemen et al. (2019)
28	*Benkara malabarica*	Scopoletin	Roots extract	Strychnine and INH induced	Swiss albino mice	25 mg/kg and 50 mg/kg	inhibitor of GABA-T	Mishra et al. (2010)
29	*Canarium sweinfurthii*	Alkaloids, flavonoids, saponins, and a variety of terpenes (monoterpenes, triterpenes, carotenoids, sesquiterpenes, cyclohexanes, and sterols), tannins, and steroids	Stem barks extract	MES, 4-AP, PTZ induced	Swiss albino mice	11.9 mg/kg	Increased GABA levels and decrease GABA-T	Kandeda et al. (2021c)
30	*Satchys Lavandulifolia*	Phenylethanoid, terpenoid, and flavonoid	Aerial parts extract	PTZ induced	Mice	50 mg/kg	Modulation of benzodiazepine receptor of GABAA	Nia et al. (2022)
31	*Sambucus nigra*	Anthocyanins, vitamins, calcium and iron, tannins, sterols	Aerial parts extract	PTZ and MES induced	Mice	250, 500 and 1,000 mg kg-	Increased GABA levels	Ataee et al. (2016)
32	*Valeriana edulis*	Valepotriate fraction	Roots extract	PTZ induced	Wistar rats	100 mg/kg	Enhanced expression of GABAAR	González-Trujano et al. (2021)
33	*Morus nigra*	Prenyl flavonoid (Morusin)	Fruits extract	Strychnine induced	Albino Wistar mice	125, 250, and 500 mg/kg	increasing the GABA levels	Zehra et al. (2021)
34	*Magnolia officinalis*	Magnolol and Honokiol	Whole plant extract	PTZ and EKP induced	Zebrafish	200 mg/kg	increasing the GABA levels	Li et al. (2020)
35	*Decalepis nervosa*	Alkaloids, flavonoids, glycosides, Quercetin and Gallic acid and phenols	Roots extract	PTZ and INH induced	Mice	250 and 500 mg/kg	increasing the GABA levels	Das et al. (2022)
36	*Scutellaria baicalensis Georgi*	Baicalin	Phytochemical	PILO induced	Sprague-Dawley rats	100 mg/kg	Enhanced GABAAR	Liu et al. (2012)
37	*Lantana camara L*	Ursolic acids stearoyl glucoside (UASG)	Whole plant extract	MES and INH induced	Wistar albino rats	50 mg/kg	Increasing GABA levels and inhibiting GAD	Kazmi et al. (2012)
38	*Artemisia indica Linn*	Ursolic acid, and oleanolic acid	Whole plant extract	PTZ induced	Swiss mice	10, 30, and 100 mg/kg	Interact with GABAA receptors via the benzodiazepine binding site	Khan et al. (2016)
39	*Dennettia tripetala G*	1-nitro-2-phenylethane	Leaf, fruit and seeds oil	PTZ induced	Mice	(50–400 mg/kg	Increasing inhibitory effects of GABA	Oyemitan et al. (2013)
40	*Hippeastrum vittatum*	Montanine	Phytochemical	PTZ induced	Swiss albino mice	60 mg/kg	Modulation of BDZ site of the GABA receptor	Da Silva et al. (2006)
41	*Eucalyptus citriodora Hook and Zanthoxylum schinifolium*	Isopulegol	Phytochemical	PTZ induced	Swiss mice	100 and 200 mg/kg	Positive modulation of GABAA receptors	Silva et al. (2009)
42	*Carum carvi*	Epoxycarvone	Phytochemical	PTZ and MES induced	Swiss albino mice	300 mg/kg	Positive modulation of GABAA receptors	Salgado et al. (2015)
43	*Erythrina mulungu*	Erysothrine	Flowers extract	Bicuculline, PTZ, and KA induced	Wistar rats	0.001–10 μg/mL	GABA modulation	Rosa et al. (2012)
44	*Nectandra grandiflora*	(+)-Dehydrofukinone (DHF)	Phytochemical	PTZ induced	Swiss mice	10, 30 and 100 mg/kg	Positive GABAergic neuronal inhibition	Garlet et al. (2017)
45	*Scutellaria baicalensis*	Wogonin	Phytochemical	PTZ, MES, and Strychnine induced	Sprague-Dawley rats	5 and 10 mg/kg	Modulation of GABAergic neuron	Park et al. (2007)
46	*Passiflora sp*	Vitexin	Phytochemical	PTZ induced	Wistar rats	100 and 200	GABAA–benzodiazepine receptor modulation	Abbasi et al. (2012)
47	*Nepeta sibthorpii Bentham*	Ursolic acid	Phytochemical	PTZ induced	Swiss mice	2.3 mg/kg	Modulation of GABAergic system	Taviano et al. (2007)
48	*Ginkgo biloba L*	Bilobalide	Phytochemical	4-AP induced	ddY strain mice	30 mg/kg	Inhibition of GAD activity	Sasaki et al. (2000)
49	*Origanum, Satureja*	Carvacrol	Phytochemical	PTZ and MES induced	Swiss mice	50, 100, and 200 mg/kg	GABAergic neurotransmitter system	Quintans-Júnior et al. (2010a)
50	*Crocus sativus L*	Safranal	Phytochemical	PTZ induced	Wistar rats	145 mg/kg	Interaction GABAA-benzodiazepine receptor complex	Hosseinzadeh and Sadeghnia, (2007)
51	*Harungana madagascariensis*	Flavonoids, alkaloids, phenols, glycosides, saponins, terpenoids and steroids	Leaves extract	INH induced	Mice	100, 500, 11,000 mg/kg	Increase GABA levels	Nnamdi et al. (2022)
52	*Malvaviscus arboreus*	Phenolic acids, β-resorcylic, caffeic, protocatechuic, and 4-hydroxyphenylacetic acids, in addition to two flavonoids; trifolin and astragalin	Whole plant extract	PTZ, PIC and Strychnine induced	*Mus musculus* Swiss	122.5, 245 and 490 mg/kg	Interact with GABAergic system	Adassi et al. (2023)
53	*Ipomoea asarifolia*	Alkaloids, cardiac glycosides, flavonoids, saponins, tannins, triterpenes and steroids (	Leaf extract	PTZ and MES induced	Swiss albino mice	300 mg/kg	Interact GABAergic pathway	Chiroma et al. (2022a)
54	*Biophytum umbraculum Welw. Syn*	Flavonoids, saponins, tannins, steroids, phenols, and terpenoids	Roots extract	MES and PTZ induced	Swiss albino mice	100, 200, and 400 mg/kg	increase in GABAergic neurotransmission	Fisseha et al. (2022)
55	*Urtica dioica Linn*	Steroids, terpenoids, flavonoids specially quercetin, isoquercitrin, astragalin, kaempferol, isorhamnetin and rutin, phenolics, i.e., phenylpropanes, scopoletin, caffeic acid and chlorogenic acid, coumarins, polysaccharides, proteins, lectins, vitamins and minerals	Roots extract	MES and PTZ induced	Swiss albino mice	100 and 200 mg/kg	modulating the (GABA) receptor-Cl- channel complex	Loshali et al. (2021)
56	*Pentas schimperiana*	Tannins, alkaloids, terpenoids, flavonoids, steroids, phenolic compounds, and proteins	Roots extract	MES and PTZ induced	Swiss albino mice	100, 200, and 400 mg/kg	modulate GABA-mediated chloride channels	Fisseha et al. (2021)
57	*Dennettia tripetala*	Alkaloidal compounds (uvariopsine, Stephan thrine, argintinine), vanillin, tannins, steroids, flavonoids, cardiac glycosides, saponins, and terpenoids	Seeds extract	PTZ induced	Swiss albino mice	61.25, 122.5 and 245 mg/kg)	enhancement of GABAergic activity	Uruaka and Georgwill, (2020)
58	*Bambusa vulgaris*	Alkaloidal compounds (uvariopsine, Stephan thrine, argintinine), vanillin, tannins, steroids, flavonoids, cardiac glycosides, saponins, and terpenoids	Leaves extract	PTZ induced	Mice	100, 200 and 400 mg/kg	GABAA-benzodiazepine receptor neurotransmission	Adebayo et al. (2020)
59	*Artemisia afra*	Alkaloids, tannins, flavonoids, and phenolic compounds	Whole plant extract	PTZ induced	BALB/c mice	250, 500, and 1,000 mg/kg	Enhanced GABA transmission	Kediso et al. (2021)
60	*Psychotria camptopus Verdc*	Flavonoids: Rutin and Butin; two triterpenoid saponins Psycotrianoside B and Bauerenone and four alkaloids 10-Hydroxy-antirhine, 10-hydroxy-iso-deppeaninol, Emetine and Hodkinsine	Trunk bark extract	Strychnine, PIC, and thiosemicarbazide induced	Wistar rats	40, 80 and 120 mg/kg	Interact with benzodiazepine site of GABAA receptors	Fokoua et al. (2021b)
61	*hrysanthellum americanum (L.)*	Alkaloids, anthraquinones, flavonoids, glycosides, phenols, glycosides, saponins, sterol, coumarins and tannins	Whole plant extract	PTZ induced	Mice	27.69, 69.22, 138.45, 276.9 mg/kg	Activation of GABAA complex receptor	Nguezeye et al. (2023)
62	*Combretum lanceolatum*	Flavonoids, lignan triterpenoids, non-protein amino acids, phenolic compounds and tannins	Leaves extract	PTZ induced	Zebrafish	01–10	interaction with the GABAA receptor	da Silva et al. (2022)
63	*Detarium senegalense*	Tannins, saponins, alkaloids, flavonoids, terpenoids, steroids, anthraquinones, glycosides, reducing sugars and resins	Leaves extract	PTZ, Brucine, and INH induced	Swiss mice	100, 200, and 400 mg/kg	Modulation GABA receptors	Nwachukwu et al. (2022)
64	*Calotropis procera*	Morphine, digoxin, quinine, and atropine	Leaves extract	Strychnine, PIC, PTZ, and PILO induced	ICR mice	100–300 mg/kg	Interaction Benzodiazepine site of GABARs	Obese et al. (2021)
65	*Gastrodia elata B1*	Vanillin	Roots extract	PTZ, 4-AP induced	Sprague-Dawley rats	25 and 50 mg/kg	Inhibition GABA transaminase	Ha et al. (2000)
66	*Musa paradisiaca*	Alkaloids, saponins, tannin and flavonoids	Stem extract	PTZ induced	Albino rats	75% (v/v)	Increasing GABA levels and inhibiting GABA-T	UGWUOKE et al. (2023)
67	*Sarcostemma acidum*	Alkaloids, flavonoids, phenolic compounds, and steroids	Aerial parts extract	MES and Phenobarbitone	Albino mice	(200 and 400 mg/kg	Increasing GABA transmission	Parmar et al. (2022)
68	*Alchemilla Kiwuensis Engl*	Flavonoids, alkaloids, and tennins	Whole plant extract	PTZ induced	Albinos Wistar rats	40 mg/kg, 80 mg/kg	Inhibiting GABA-Transaminase	Foutsop et al. (2023)
69	*Cocos nucifera L*	Flavonoids, alkaloids, phenols	Flowers extract	MES and PTZ induced	Wistar rats	125, 250, and 500 mg/kg	Elevated level of GABA	Archana et al. (2021)
70	*Paullinia pinnata*	Flavonoids, tannins, other phenolic compounds; and alkaloids	Leaves extract	INH induced	Swiss albino mice	100, 200, and 400 mg/kg	Increase GAD activity and decrease GABA-T	Ajibade et al. (2022)
71	*Afzelia africana*	Alkaloids, saponins, steroids, tannins, cardiac flavonoids, and glycosides	Leaves extract	PILO induced	Wistar rats	100 mg/kg	Interact with BZD site on the GABAAR	Kinsou et al. (2019)
72	*Boswellia dalzielii*	Saponins, tannins, steroids/terpenoids and flavonoids	Stem extract	PTZ and MES induced	Swiss albino mice	500 mg/kg	Modulation of GABAAR	Medugu et al. (2020)
73	*Amaranthus spinosus*	alkaloids, anthraquinones, saponins, cardiac glycosides, tannins, flavonoids, carbohydrates, and triterpenes	Leaves extract	PTZ induced	Albino mice	400 and 800 mg/kg	Enhanced GABA mediated inhibitory neurotransmission	Usman et al. (2023)

Secondly, the extracts and phytochemicals can also interact with the GABAAR. GABAAR is also a ligand-gated channel, and GABA neurotransmitters act as a ligand. When GABA neurotransmitters bind with the GABAAR on postsynaptic neurons, that leads to the opening of chlorine channels and, finally, the influx of Cl^−^ into the cell, which reduces neuronal excitability by hyperpolarization of the membrane (Ghit et al., 2021). Dysfunction or mutation in the subunit(s) of GABAAR fails to hyperpolarize the membrane (Mele et al., 2019). Several studies have reported the interaction of phytochemicals and plant extracts with GABAAR, causing hyperpolarization and reducing neuronal excitability. Arenaria kansuensis Maxim. (AKM) and Asterothamnus centrali-asiaticus Novopokr. (ACN) are considered the two important medicinal herbs worldwide, especially in Chinese medicine, Persian, ancient Greek, Central Asian, and Ayurved. The phytochemicals analysis of these plants showed that they are rich in alkaloids and flavonoids and possess various medicinal properties (Wang et al., 2016a; Wang et al., 2016b). Liu et al. investigated the structure-activity relation of flavonoids isolated from these plants with the benzodiazepine site of GABAAR and searched for anticonvulsive compounds in the PTZ-induced epilepsy mice model. They revealed that several flavonoids isolated from the whole plant extract of these plants had a strong binding affinity with GABAAR. In addition, 2′,4′,5,7-tetrahydroxy-5′, 6-dimethoxyflavone, a flavonoid compound isolated from the ACN, exhibited potent anticonvulsant activity. Finally, the authors concluded that flavones could be considered strong antiepileptic drugs that have a strong affinity for the GABAAR receptor and can be used for the treatment of seizures (Liu et al., 2018). However, further studies are required to determine the safety, further efficacy, side effects, and dosage of the extract or isolated phytochemical flavone. Similarly, the interaction of Euterpe oleracea Martius (Açai) *extract* with GABAAR has also been reported (Muto et al., 2022). HPLC/MS analysis of Acai revealed that Açai stone possesses high levels of various polyphenolic compounds such as caffeic acid, cinnamtannin, procyanidin, catechin, polymeric proanthocyanidins, followed by traces of another phenolic compound (Muto et al., 2021). In this study, the authors used PTZ-induced epileptic rats as an epilepsy model and used a specific dose of 300 mg/kg and compared with the marketed drug diazepam and evaluated by electroencephalographic (EEG) profiling. The authors reported that there is no significant difference between the experimental group and the treatment group with diazepam. They further conclude that *Euterpe oleracea* stone (EEOS) extract interacts with the benzodiazepine subunit of GABAAR and exerts anticonvulsive activity. Therefore, this extract could also be used to treat epilepsy. However, extensive investigation could be required on the safety and further efficacy. Similarly, several studies have been reported on the interaction of plant extract and phytochemicals with GABAAR, which could alleviate or treat seizures and epilepsy, summarized in Table 1.

The third possible mechanism through which the extract or phytochemicals interact with the inhibitory pathway is the targeting of GABA-T. In the pre-synaptic neurons, the GABA-T converts the GABA into succinic semialdehyde (SSA) and, therefore, makes the neuron GABA deficient and elevates the glutamate levels, which are excitatory neurotransmitters (Lu et al., 2023). It has been reported that various phytochemicals and bioactive molecules from various medicinal plants can interact with this enzyme, leading to increased GABA levels and decreased glutamate levels to balance the excitatory and inhibitory potential, hence alleviating seizure. Mishra et al. investigated the anticonvulsive effect of Benkara malabarica (Linn.) *ethanolic roots extract in* strychnine-induced and INH-induced acute convulsion mice model (Mishra et al., 2010). This is a medicinal plant and is commonly used in various regions of India for the treatment of various types of diseases, including epilepsy (Nadkarni, 1954). The authors used 2 mg/kg for strychnine and 300 mg/kg for the INH model and compared with the group treated with standard drugs like phenytoin and diazepam. The authors reported that the plant extract has strong antiepileptic properties and could be used as a treatment option after further investigation. The authors hypothesized that the antiepileptic activities of the plant extract are due to the interaction of phytochemicals with GABA-T. To prove this hypothesis, the authors performed a GABA-T activity assay as described by Salvador and Albers (Salvador and Albers, 1959). The authors documented that the plant extract had GABA-T inhibitory activity (IC_50_ = 0.721 mg/mL). In addition, the GABA-T inhibitory activity of Scopoletin, which is a major constituent of the extract, was IC_50_ = 10.57 μM. They further concluded that GABA-T inhibitory activity might be due to scopoletin alone or in combination with other compounds in the extract.

Similarly, another study has reported that the aqueous stem bark extract of *C. schweinfurthii* alleviates seizures (Kandeda et al., 2021c). They also reported that the GABA pathway is involved. The authors used various doses against different animal models of epilepsy and reported that the different doses of extract showed significant antiepileptic activity against various animal models. Further studying the mechanism of action of the extract, they concluded that the phytochemicals in the plant extract interacts with the GABA pathway as after treatment with the extract, the GABA levels increased while the GABA-T levels were reduced. They concluded that the antiepileptic activity of *Canarium schweinfurthii* was due to the inhibition of GABA-T by the bioactive compounds present in the extract, which could be used alone or in combination with other drugs for seizure control. Furthermore, this plant has a double mechanism of action (increased GABA and decreased GABA-T); therefore, it could be a strong AED in the near future after further investigation. Several other studies have been reported on the interaction of extracts and various phytochemicals with the GABA-T, as summarized in Table 1.

## 5 Modulation of excitatory pathway

Glutamate is one of the principal excitatory neurotransmitters in the CNS that plays a critical role and performs various functions such as learning, memory, synaptic plasticity, nerve degeneration, neurogenesis, and many more (Haroon et al., 2017). However, excessive glutamate in the body leads to severe pathogenic events that may then cause diseases such as Alzheimer’s disease, Parkinson’s, and epilepsy. The receptors for glutamate are broadly categorized into two classes, i.e., ionotropic glutamate and metabotropic receptors (Afshari et al., 2020). The ionotropic receptors include NMDA (Ca^2+^ influx), kainate, and AMPA (Na^+^ Influx) receptors, while the latter one includes G protein-coupled receptors that activate the intracellular cascade (Crupi et al., 2019). Among these receptors, the NMDA and AMPA play a significant role in the initiation and propagation of epileptic seizure as these both allow the high influx of Ca^+^ and Na^+^ ions, respectively, which leads to the imbalance of excitatory and inhibitory pathways and therefore, these two receptors represent key clinical research targets (Hanada, 2020). It is well known that the agonists for these two receptors lead to the development of epilepsy in animals or humans. While antagonist leads to the inhibition of seizures by blocking respective receptors, and therefore, these are potential targets for the development of drugs against epilepsy. Several studies have reported that medicinal plants and phytochemicals can also lead to the modulation and inhibition of these receptors, which may lead to the development of potent drugs against epilepsy.

**FIGURE 3:**
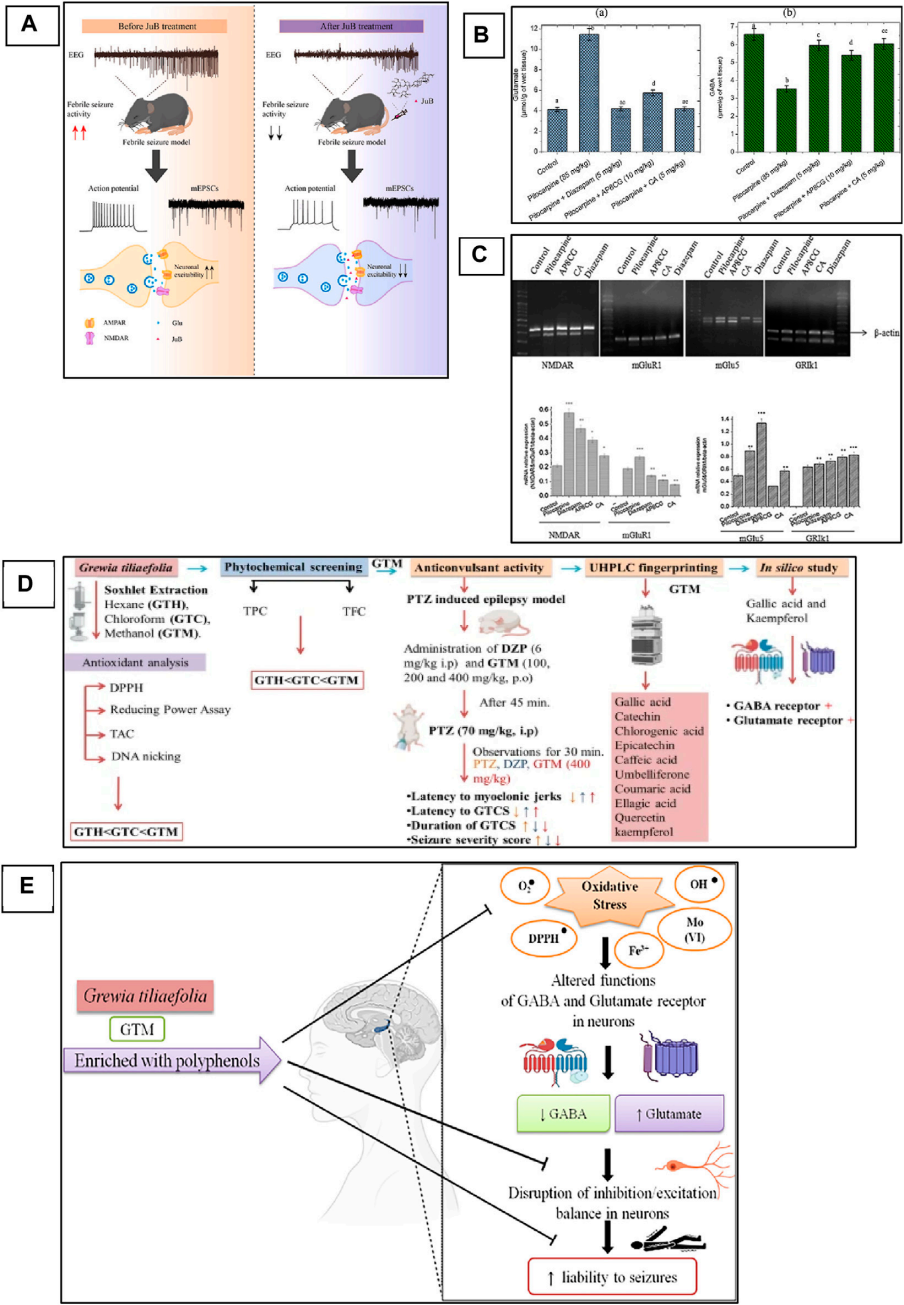
Phytochemicals can modulate NMDAR and AMPAR in the animal model of epilepsy. **(A)** Mechanistic overview of the effect of Jujuboside B from medicinal plants against febrile seizures and the mechanism of action on AMPA receptor (Jin et al., 2023). Copyright permission@ Elsevier 2023. **(B)** apigenin-8-C-glycoside (AP8CG) and Chlorogenic acid (CA) effects on the levels of glutamate and GABA in the hippocampus of mice and their comparison with standard drugs and control group. **(C)** mRNA profile of NMDAR, mGlu1, and mGLU5 in experimental groups (treated with AP8CG and CA and control groups (Aseervatham et al., 2016). Copyright permission@ Elsevier 2023. **(D)** A mechanistic overview of the antiepileptic activity of Grewia tiliaefolia in mice followed by *in silico* analysis of important phytochemical that involved in the modulation of NMDAR. **(E)** Possible antiepileptic mechanism of Grewia tiliaefolia (Rajput et al., 2023). Copyright permission@ Springer Nature 2023.

*Zizyphi Spinosi Semen* (ZSS) is a medicinal plant widely used in Chinese medicine for the treatment of various diseases, including insomnia and some other psychiatric disorders (Zhao et al., 2016). The phytochemical analysis of this plant has reported that it contains various types of bioactive compounds that have strong medicinal values and are used for the treatment of various diseases. Several saponins have been identified from ZSS, including seven types of jujuboside (A-H) (Xiao et al., 2018; He et al., 2020). Xi Wang reported that Jujuboside A has strong antiepileptic activities by increasing the levels of GABA in the hippocampus, which are inhibitory neurotransmitters (Wang et al., 2015). Similarly, Panpan Song et al. reported that jujuboside B (jub B) also leads to an increase of the GABA as well as overexpression of GABAAR, which plays a critical role in the control of seizures. A febrile seizure is a non-epileptic seizure affecting children between 3 months and 5 years, and phytotherapeutic treatment could also help in the control of this seizure by targeting the excitatory pathway of the CNS (Song et al., 2017; Jin et al., 2023 hypothesized and then proved that jujuboside can modulate the excitatory pathway by inhibiting the glutamate receptor AMPA and could be used as a potent agent against febrile seizure (Jin et al., 2023). In this study, they used EEG recording for the monitoring of current in the mouse model of febrile seizure and to evaluate the efficacy of the Jub B as shown in Figure 3A. The mass spectroscopy was used for the identification of Jub B in the brain, and they revealed that significant amount of Jub B was present in the brain, it means that it can easily cross the BBB which is one the main barrier in the development of drugs against neurological disorders. The authors then finally concluded that Jub B can suppress the neuronal excitation in the hippocampus by inhibiting the activity of AMPAR and, therefore, cause relief from febrile seizure. JuB B showed a strong antiseizure effect against epileptic seizures; however, similar targets are also involved in epileptic seizures. Therefore, it could be used against various animal models of epilepsy and could show a strong antiepileptic activity based on the above results.

Similarly, apigenin-8-C-glycoside (AP8CG), commonly known as vitexin, and Chlorogenic acid (CA), are two important phytochemicals present in various plants and widely used in medicines (Liang and Kitts, 2015). The antiepileptic activities of these two compounds have been reported in PILO-induced epilepsy mice models (Aseervatham et al., 2016). The authors reported that they administered a 10 mg/kg dose of AP8CG and a 5 mg/kg dose of CA to mice, and the results were compared with the standard drug diazepam. The authors reported that both compounds showed potential antiepileptic activities. However, CA showed stronger activities on the levels of glutamate and GABA than AP8CG, as shown in Figure 3B. By explaining the mechanism of action, the authors revealed that these compounds selectively inhibit the expression of NMDAR, Metabotropic glutamate receptor 1 (mGlu1), and Metabotropic glutamate receptor 5 (mGlu5). They also evaluated the mRNA profile of NMDAR, mGlu1, and mGLU5 and compared them with the standard drugs and control groups and reported that a significant association was observed in the experimental groups and other groups, as shown in Figure 3C. Therefore, these two compounds need further investigation and could be used for the treatment of epilepsy.

Recently, Ankita Rajput and colleagues carried out a comprehensive study on the antiepileptic activity of aerial parts extract of *Grewia tiliaefolia* in the PTZ induced mice model of epilepsy and further screening of the plant followed by *in silico* analysis of the phytochemical as shown in Figure 3D (Rajput et al., 2023). The other members of the family of this plant showed neuroprotective activities, antidepressants, anti-anxiety, etc (Jebin et al., 2019). Here in this study, the authors reported that the plant extract significantly increased the latency of myoclonic jerks and generalized tonic-clonic seizures (GTCS). In addition, it also reduced the severity of seizures associated with the GTCS. Further, they analyzed the extract with HPLC for phytochemical profile and found that the extracts have several bioactive compounds that could be used for various neurological diseases. From the *in silico* analysis of these phytochemicals, the authors revealed that gallic acid and kaempferol compounds from the extract have shown interaction as agonists with GABA and antagonists with Glu-NMDA. Therefore, they concluded that the possible antiepileptic effect of the extract is due to the increase of GABA and the decrease of Glu-NMDA. They further revealed that this effect could be due to these two compounds, and the possible mechanism can be, as shown in Figure 3E. However, further studies could be carried out to confirm the involvement of these two compounds that can be used then as a potent antiepileptic agent in the future. A similar study has been reported on the computational investigation of *W. somnifera* and their *in silico* analysis of the phytochemicals (Kumar and Patnaik, 2016). The authors analyzed 25 different phytochemicals that showed related activities and found that Anaferine, Beta-Sitosterol, Withaferin A, Withanolide A, Withanolide B, and Withanolide D have shown the interaction with GluN2B containing NMDAR, which is one of the main targets to control the seizure. Therefore, these compounds could be used *in vivo* first, and after confirmation and thorough investigation of safety and efficacy, they can be used as potent antiepileptic agents in the future.

Several studies have reported that the phytotherapeutic treatment can modulate NMDAR and AMPAR, which can alleviate seizures and epilepsy. However, there are some limitations to every study. Therefore, to develop a potent antiepileptic agent, extensive investigation could be carried out not only on the reduction of seizure and severity but also on the side effects or safety of the plant or phytochemicals. We have summarized the literature on phytochemicals and plant extracts that modulate the excitatory pathway, especially NMDAR and AMPAR, as shown in Table 2.

**TABLE 2:** The antiepileptic properties of plant extract and purified phytochemicals through the modulation of the glutamatergic pathway (especially NMDARs and AMPARs).

S. No	Plant	Phytochemical	Plant extract/Isolated phytochemical used	Model(s) used	Specie (s)	Dose (s)	Target(s)	References
1	*Artemisia persica*	Apigenin, luteolin, quercetin, ruti, caffeic acid	Whole plant extract	PTZ induced	NMRI mice	100, 200, 400 mg/k	Reduced the expression of NMDA	Nasiri-Boroujeni et al. (2021)
2	*Vitis vinifera*	Proanthocyanidin	Phytochemical	PILO, PIC, and strychnine induced	Albino mice	200, 100, 50 mg/kg	Inhibition of glutamatergic/NMDA transmission	Osuntokun et al. (2022)
3	*Ipomoea asarifolia*	*Alkaloids, cardiac glycosides, flavonoids, saponins, tannins, triterpenes and steroids*	Leaves extract	PTZ and MES induced	Swiss albino mice	75 mg/kg	Acting on glutamatergic pathway	Chiroma et al. (2022b)
4	*Grewia tiliaefolia*	Gallic acid and kaempferol	Aerial parts	PTZ induced	Mice	400 mg/kg	Antagonistic interaction for Glu-AMPA receptor	Rajput et al. (2023)
5	*Rubus idaeus*	Ellagic acid (EA)	Phytochemical	PTZ induced	NMRI mice	6.25, 12.5, and 25 mg/kg	Attenuation of the NMDA-R pathway	Rahimi-Madiseh et al. (2022)
6	*Uncaria rhynchophylla*	Rhynchophylline (RIN)	Phytochemical	PILO induced	Sprague–Dawley rats	100 μM	Alternation of NMDA currents and NR2B expression	Shao et al. (2016)
7	*Melissa officinalis L*	Alkaloids and other phytochemicals	Essential oil	PTZ induced	Adult Swiss mice	50 and 100 mg/kg	Inhibition of glutamate release	Chindo et al. (2021)
8	*Grewia tiliaefolia*	Caffeic acid, Umbelliferone, Coumaric acid, Ellagic acid, Quercetin, Kaempferol	Aerial parts extract	PTZ induced	Mice	400 mg/kg	Antagonistic interaction with Glutamate AMPA receptor	Rajput et al. (2022)
9	*Eugenia caryophyllata*	Eugenol	Essential oil	PILO induced	Sprague Dawley male rats	0.1 mL/kg	Interact with NMDA receptors	Parvizi et al. (2022)
10	*Ilex paraguariensis St. Hilaire*	Polyphenols, xanthines, cafeolyl derivatives, saponins, and minerals	Extract	KTM induced	Wistar rats	10 mg/kg	Modulation of AMPA receptors	dos Santos Branco et al. (2013)
11	*Berberis vulgaris L*	Berberine	Phytochemical	KA induced	Male albino Wistar rats	25, 50 and 100 mg/kg	Interact NMDA receptors	Mojarad and Roghani, (2014)
12	*Rosemarinus officinalis*	β-Caryophyllene	Phytochemical	PIC induced	Adult C57BL/6 mice	100 mg/kg	Modulation of glutamatergic pathway	de Oliveira et al. (2016)
13	*Mallotus oppositifolius*	*Alkaloids, cardiac glycosides, flavonoids, saponins, tannins, triterpenes and steroids*	Whole plant extract	PTZ induced	Mice	1,000–3,000 mg kg-1	Modulation of NMDA	Kukuia, (2012)
14	*Rauvolfia ligustrina Willd*	Alkaloids and flavonoids	Root extract	PTZ, PIC, and MES induced	Male Wistar rats	62,5 mg/kg	Glutamatergic neurotransmitter system	Quintans-Júnior et al. (2010b)
15	*Bacopa monnieri*	Saponins, bacosides A and B	Extract	PILO induced	Mice	1 mg/kg	Interact with the NMDA R1 gene expression	
16	*Crassula arborescens (Mill.)*	Flavonoids, tannins, reducing sugar, saponins and triterpene steroids	Leaves extract	bicuculline, picrotoxin, and PTZ induced	Mice	4,000 mg/k	Modulation of glutamatergic pathway	Amabeoku et al. (2014)
17	*Withania somnifera*	*Withanolide A (WA)*	Roots extract	PILO induced	Male Wistar rats	100 mg/kg	decreased NMDA receptor density	Soman et al. (2012)
18	*Populus deltoides*	Glycoside, resin, tannin, alkaloid, flavanoid, terpenoid, protein, saponin, anthraquinone, Coumaric, narcission, ascorbic acid, fibers, vitamins, iron, minerals, protein and amino acid	Leaves extract	MES, PTZ, and Strychnine induced	Swiss albino mice	25, 250, and 500 mg/kg	Inhibition of NMDA receptors	Kumar et al. (2017)
19	*Alchemilla Kiwuensis Engl*	Flavonoids, alkaloids, and tennins	Whole plant extract	PTZ induced	Albinos Wistar rats	40 mg/kg, 80 mg/kg	Modulation of glutamatergic pathway	Foutsop et al. (2023)
20	*Cyperus rotundus*	β -sitosterol, cyperene, cyperol, flavonoids, sesquiterpenoids, vitamins and polyphenols	Roots extract	MES and PTZ induced	Albino rats	100 mg/kg	Inhibition of NMDAR	Shivakumar et al. (2009)
21	*Psydrax subcordata (DC.)*	Anthiumosides 1–5, together with nine known compounds, shanzhigenin methyl ester, 1-epishanzhigenin methyl ester, linearin, 1-epilinearin, mussaenoside, shanzhiside methyl ester, 3′,4′,7-trihydroxyflavone, betulinic acid and oleanolic acid	Leaves extract	4-AP, PTZ, PIC, and INH induced	Mice	30, 100 and 300 mg/kg	Inhibition of glutamate mediated excitation	Daanaa et al. (2018)

^a^ For abbreviations, see List of abbreviations.

## 6 Interaction with Voltage Gated Calcium Channels

**FIGURE 4:**
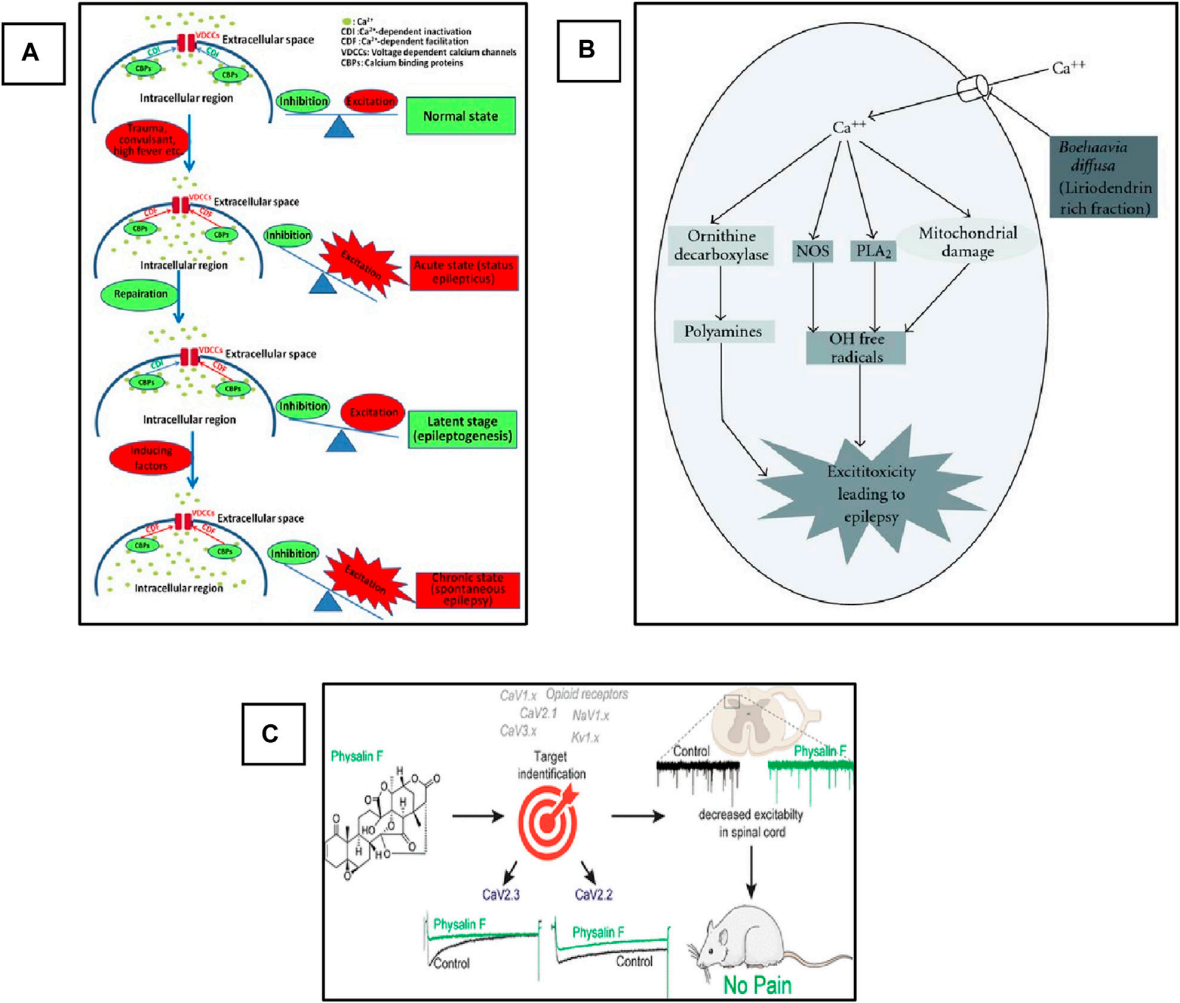
Schematic representation of the role of VGCCs in epilepsy and pharmacological targets for phytochemicals. **(A)** Role of Ca^+2^ channels in the development of epilepsy. Adopted from (Xu and Tang, 2018). Copyright permission@ MDPI 2018, **(B)** Possible anti-convulsant mechanism of **(B)**. diffusa on the inhibition of VGCCs. Adopted from (Kaur and Goel, 2011). Copyright permission@ Hindawi 2011. **(C)** Schematic representation of inhibition of CaV2.3 (R-Type) and CaV2.2 (N-Type) Voltage-Gated Calcium Channels by Physalin F in pain model (Shan et al., 2019). Copyright permission@ ACS 2019.

Voltage Gated Calcium Channels (VGCCs), also termed Voltage-Dependent Calcium Channels (VDCCs), are widely expressed in the CNS of humans and other mammalian and play a critical role in the regulation of synaptic transmission, phosphorylation/dephosphorylation of protein, gene transcription, as well as perform various other functions including the survival and death of the cell and adaptive responses to the synaptic activity (Xu and Tang, 2018). However, alteration in these channels leads to the imbalance of cellular events that causes pathological consequences (Djamshidian et al., 2002). The abnormal activation of these channels leads to the influx of Ca^2+^ into the cell, which plays a critical role in the imbalance of the action potential and finally helps in the triggering and propagation of seizure. Based on the electrophysiological studies, the VGCCs are classified into five different types, i.e., L-, N-, P/Q-, R-, and T-type (Catterall, 2000). The detailed explanation of VGCCs regarding structure, function, types, and role in the pathophysiology of epilepsy has been comprehensively explained by Jie and Feng, as shown in Figure 4A (Xu and Tang, 2018), and Rajakulendran and Michael. Several clinically approved drugs target these channels for the control of seizures and the treatment of epilepsy. Similarly, medicinal plants have diverse bioactive compounds that can effectively block these channels and can control seizures. Currently, various studies on the use of plant extracts and phytochemicals against epilepsy targeting VGCCs have been reported. We suggest that if extensive investigations of these phytotherapeutics are carried out to determine their safety, efficacy, and dosage, then these could be used as potential agents for the treatment of epilepsy.

Mandeep and Rajesh evaluated the anticonvulsive activity of dried root extract of Boerhaavia diffusa (BD) in the PTZ-induced model of Swiss albino mice (Kaur and Goel, 2009). BD contains several phytochemicals that have a potential medicinal value, including flavonoids, terpenoids, punarnavoside, Liriodendrin, liriodendrin, and many more (Sahu et al., 2008). At the end of the 20th century, it was reported that the liriodendrin isolated from the methanolic extract of BD showed antagonistic activity for calcium channels (Lami et al., 1990; Lami et al., 1991). In addition, the root extract of BD can be used for the treatment of epilepsy (Adesina, 1979). Therefore, the authors decided to investigate the anticonvulsive activity of whole root extract, phenolic compounds, and liriodendrin in various doses and compared the results with the standard drug diazepam-treated group. The authors reported that more than 80% protection and recovery were observed in the group treated with BD crude extract and liriodendrin. The authors concluded this activity could be due to the antagonistic activity of the liriodendrin, and they designed a mechanistic pathway of the possible mechanism of the plant extract against VGCCs, as shown in Figure 4B. Briefly, the authors concluded that the observed anti-convulsant activity was attributed to its VGCCs antagonistic action. This activity was retained only in the liriodendrin-rich fraction isolated from *Boerhaavia diffusa*. Additionally, this was confirmed by the significant anti-convulsant activity of the liriodendrin-rich fraction in BAY k-8644-induced seizures. In addition to extracts and fractions having antiepileptic activity, they act on i) other ionic channels such as sodium and potassium, ii) antioxidant activity, iii) neurotransmitter modulation, and iv) anti-inflammatory activity (Kaur and Goel, 2011).

Recently, a very interesting study carried out by Paz and colleagues used a natural product, Argentina C, derived from the native American medicinal plant species *Parthenium incanum* against mouse model of postoperative pain (Duran et al., 2023). The authors reported that this compound attenuates the pain by inhibiting the two most important VGSCs and VGCCs. They concluded that the compounds have very strong dual inhibiting properties and could be used for the treatment of pain. On the other hand, these two channels also play a significant role in the pathophysiology of epilepsy and are two important pharmacological targets in epilepsy. In addition, drug-resistant epilepsy is mostly treated by a combination of two drugs with different mechanisms of action. Here, this plant-derived compound targets and inhibits two different channels. Therefore, if this compound is used against various epileptic models, especially drug-resistant models, it could show strong activity against seizure and can be used as a potent antiepileptic agent in the future. Similarly, another natural compound, Physalin F, isolated from the aerial parts extract of medicinal herb, *Physalis acutifolia*, has been used against neuropathic pain. They also reported that this compound blocks CaV2.3 (R-type) and CaV2.2 (N-type) VGCCs and decreases excitation in the spinal card, as shown in Figure 4C (Shan et al., 2019). This compound can also be used against epilepsy in animal models, and it could give promising results as these VGCCs are also the main targets in the development of drugs against epilepsy. In addition, another study reported the use of Betulinic acid (BA) derived from lavender *Hyptis emoryi* and reported that it attenuates nerve injury-associated peripheral sensory neuropathy by inhibiting the N and T-type calcium channels, which are also the main target of epileptic drugs. The results indicated that the BA leads to the downregulation and inhibition of the Cav3.2 and Cav3.3 calcium channels (Bellampalli et al., 2019). BA could also be evaluated against epilepsy and seizure. As here in this study, it showed promising results in neuropathy.

Several studies have been reported on the inhibitory effect of various types of plant extracts and derived compounds in various diseases. These compounds could be investigated against epilepsy. We summarized the details of some studies that reported various medicinal plants and phytochemicals for the treatment of seizures and epilepsy, as shown in Table 3.

**TABLE 3:** Anticonvulsive effect of plant extract and phytochemicals isolated from various plants targeting VGCCs, VGSCs, and Kv.

S. No	Plant	Phytochemical	Plant extract/Isolated phytochemical used	Model(s) used	Specie (s)	Dose (s)	Target(s)	References
1	*Globimetula braunii*	Saponins, anthraquinones carbohydrates, flavonoids, steroids, and tannins	Leaf Extract	PTZ induced	Swiss albino mice	150 mg/kg	Inhibition of T-type Ca2+ channels	Aliyu et al. (2014)
2	*Culcasia falcifolia*	Alkaloids, polyphenols, flavonoids, glycosides, saponins, and tannins	Leaf Extract	PTZ induced	Mice	200 and 400 mg/kg	Reducing the T-type of Ca^2+^ currents	Gracelyn Portia et al. (2018)
3	*Annona senegalensis Pers*	Flavonoids, terpenoids, and diterpenoids	Root barks extract	MES induced	Male Wistar rats	1, 10, 100 and 300 µM	Targets VGSCs	Almamy et al. (2021)
4	*Pseudospondias microcarpa*	Flavonoids, saponins, phenols, terpenoids, coumarines, and cardiac glycosides	Stem bark extract	PTZ, PIC, PILO, picrotoxin and 4-AP induced	-	30, 100 and 300 mg/kg	Activated voltage dependent K^+^ channels	Wabo et al. (2009)
5	*Ageratum Conyzoides L*	Tannins, alkaloids, and small amount of reducing sugars, flavonoids, anthocyanins, steroids and terpenoids	Leaves extract	PIC and MES induced	Swiss albino mice	200, 400 and 800 mg/kg	Blocking Na^+^ channels	Viswanatha et al. (2016)
6	*Imperata cylindrica (L.)*	Flavonoids, polyphenols, and chromones (1 and 2)	Whole plant extract	Mechanical stress	*D. melanogaster*	0.1–0.5 g/mL	Voltage-gated sodium ion channels’ inhibitory properties	Ssempijja et al. (2023)
7	*Eclipta alba (linn.)*	Alkaloids, carbohydrates, phenolic compounds and coumarins	Leaves extract	MES induced	Mice	50 mg/kg	Inhibition of voltage gated sodium channels	Shaikh et al. (2012)
8	*Ipomoea asarifolia*	Flavonoids and saponins	Leaves extract	4-AP induced	Chicks (cockerel) and Swiss mice	300 mg/kg	Interfering with potassium channels	Chiroma et al. (2022a)
9	*Urtica dioica Linn*	Steroids, terpenoids, flavonoids specially quercetin, isoquercitrin, astragalin, kaempferol, isorhamnetin and rutin, phenolics, i.e., phenylpropanes, scopoletin, caffeic acid and chlorogenic acid, coumarins, polysaccharides, proteins, lectins, vitamins and minerals	Root extract	MES and PTZ induced	Swiss albino mice	100 and 200 mg/kg	inhibition of Na^+^ channels	Loshali et al. (2021)
10	*Phyllanthus Amarus*	Phyllathin	Arial parts extract	PTZ induced	Male Swiss albino mic	50, 100, and 200 mg/kg)	Inhibition of voltage-gated ion channels (Na^+^, K^+^/Ca^+2^-ATPase)	Tao et al. (2020)
11	*Hippophae rhamnoides*	Various phytochemicals	Whole plant extract	Intracortical iron (5 μL of 100 mM FeCl3) injection	Male adult Wistar rats	1 mL/kg	Modulation of Na^+^, K^+^ ATPase activity, and regulate the expression of sodium channel Nav. 1.1 and Nav 1.6	Ladol and Sharma, (2021)
12	*Ageratum Conyzoides L*	Flavonoids, alkaloids or terpenes	Whole plant extract	MES and PIC induced	Male Swiss albino mic	200, 400 and 800 mg/kg	Blocking Na^+^ or K^+^ channels	Randrianavony et al. (2020)
13	*Magnolia officinalis*	Magnolol	Phytochemical	PTZ induced	-	-	Inhibition of both VGSC and Kv channels	Gong et al. (2012)
14	*Wedelia chinensis*	Alkaloids, flavonoids, saponins, and steroids	Whole plant extract	MES and PTZ	Swiss albino mice	250, 500 and 750 mg/kg	Sodium channel blockage	Mishra et al. (2011)
15	*Carum copticum Benth*	Thymol	Phytochemical	MES, PTZ, and 4-AP induced	Adult male Wistar rats	5–25 mg/kg	Inhibition of voltage-gated Na^+^ channels	Sancheti et al. (2014)
16	*Cassia auriculata*	Flavonoids, tannins, lipids, polyphenols, triterpenoids and steroids	Seed extract	PTZ and MES induced	Mice	1000 mg/kg	Sodium channel blockage	Nanumala et al. (2018)
17	*Sapindus Emarginatus* and *Acorus calamus*	Alkaloids, flavonoids, saponins and tannins	Leaves extract	MES and PTZ induced	Albino mice	200 and 400 mg/kg	Inhibition of voltage-dependent sodium and calcium currents	SHOUGRAKPAM et al. (2021)
18	*Cyperus rotundus*	Contain β -sitosterol, cyperene, cyperol, flavonoids, sesquiterpenoids, vitamins and polyphenols	Roots extract	MES and PTZ induced	Albino rats	100 mg/kg	Inhibition of voltage gated Na^+^ channels	Shivakumar et al. (2009)
19	*Pandanus odoratissimus Linn*	Alkaloids, lipids, terpenes, triterpenoids, flavonoids and coumarins	Leaves extract	MES, PTX, and strychnine induced	Swiss Albino mice	100 and 200 mg/kg	Activation of potassium ion channels	Adkar et al. (2014)
20	*Carissa edulis*	saponins, tannins, flavonoids, and cardiac glycosides	Root bark extract	Strychnine, MES, PIC, INH, and PTZ induced	Swiss Albino mice	5,000 mg/kg	Inhibiting voltage gated sodium channels	Ya’u et al. (2015)
21	*Marsilea quadrifolia Linn*	Alkaloids, Saponins, Phenolic compounds and tannins	Whole plant extract	MES and PTZ induced	Male Wistar rats	200, 400, and 600 mg/kg	Inhibiting voltage gated sodium channels	Snehunsu et al. (2013)
22	*Solanum sisymbriifolium Lam*	Solasodine	Fruits extract	MES, PIC, and PTZ induced	Wistar albino rats	25, 50, and 100 mg/kg	Inhibition of voltage-dependent Na + channels	Chauhan et al. (2011)
23	*Psydrax subcordata (DC.)*	Anthiumosides 1–5, together with nine known compounds, shanzhigenin methyl ester, 1-epishanzhigenin methyl ester, linearin, 1-epilinearin, mussaenoside, shanzhiside methyl ester, 3′,4′,7-trihydroxyflavone, betulinic acid and oleanolic acid	Leaves extract	4-AP, PTZ, PIC, and INH induced	Albino mice	30, 100 and 300 mg/kg	Activation of potassium ion channels	Daanaa et al. (2018)
24	*Jatropha curcas*	alkaloids, tannins, saponins, carbohydrates, anthraquinones, and other phenolic compound	Leaves extract	MES induced	Male albino mice	200 and 400 mg/kg	Inhibit voltage. Dependent sodium channels	Bolanle et al. (2018)
25	*Anogeissus latifolia (Roxb.)*	Ellagic acid	Stem bark extract	MES and PTZ induced	Swiss albino mice	200, 400, and 600 mg/kg	Inhibition of voltage-gated Na + channel	Sharma and Kaushik, (2018)
26	*Anisomeles malabarica*	Flavonoids and other important phytochemicals	Leaves extract	PTZ and MES induced	Male Wistar rats	200 and 400 mg/kg	Inhibition of voltage-gated Na + channel	Choudhary et al. (2011)

## 7 Modulation of the voltage-gated sodium channels

**FIGURE 5:**
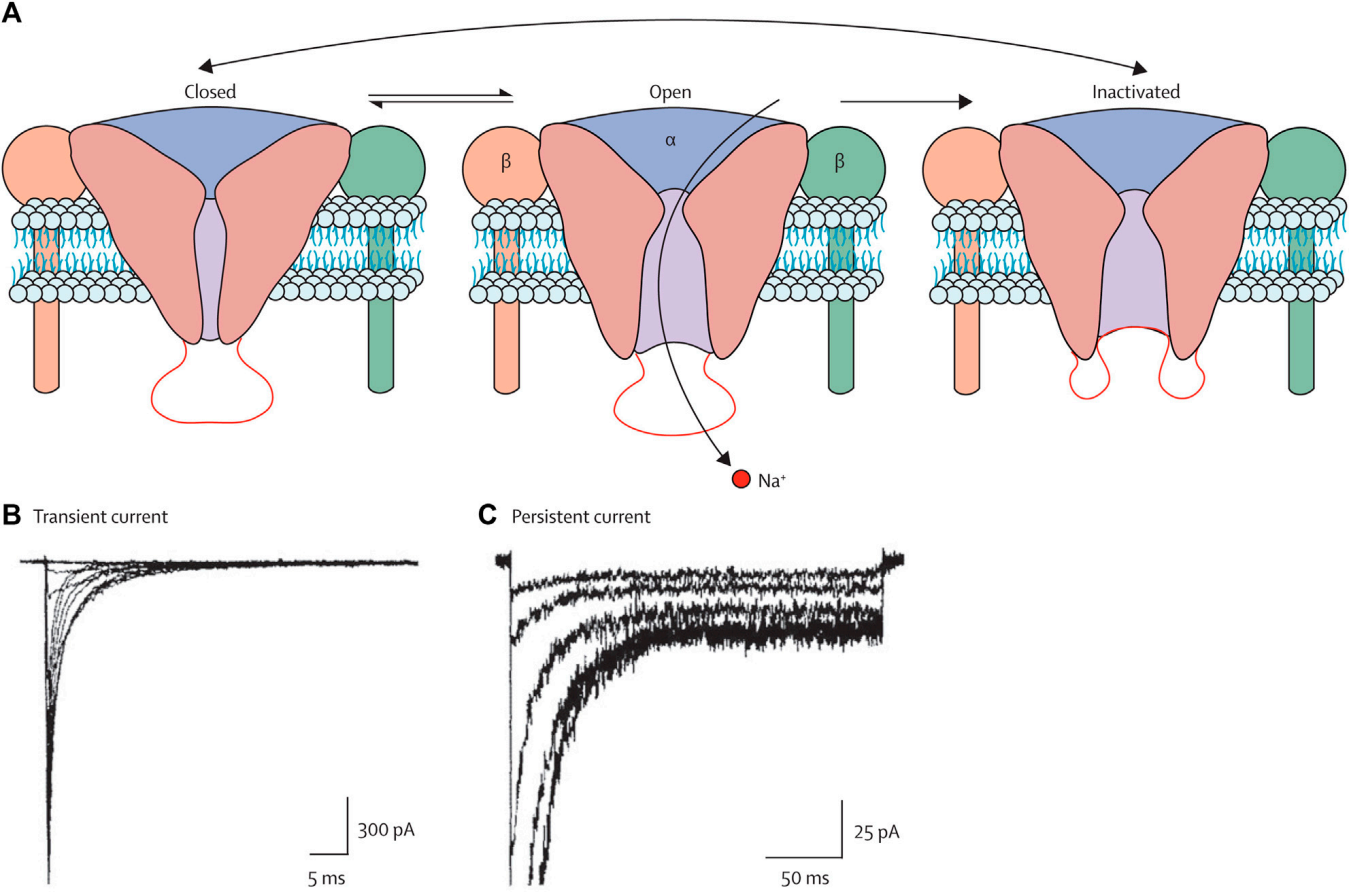
Three states of the VGSCs, i.e., closed, open, and inactivated states (Mantegazza et al., 2010). Copyright permission@ Elsevier 2010.

Voltage-gated sodium channels (VGSCs or NaVs) are membrane proteins that selectively conduct Na^+^ across the membrane. These channels are the mediators of intrinsic neuronal excitability and are responsible for the generation and propagation of action potential (Kwong and Carr, 2015). However, the abnormal expression and function of these channels lead to neurological disorders such as migraine, epilepsy, neuropathy, and pain. VGSCs have three functional states, as shown in Figure 5 (Mantegazza et al., 2010). During the resting membrane stage of the neuron, these are in the closed state. It opens in a few hundred microseconds in response to membrane depolarization by the process called activation and allows the influx of Na + to the cell, which is then inactivated in a few milliseconds by the process termed fast inactivation. The time from the activated state to the inactivated state is the most important, and the activation of channels for a longer time or longer time required from the activated state to the inactivated state is the main cause of imbalance in excitatory and inhibitory pathways that leads to epilepsy and other neurological disorders. The inhibition or blocking of VGSCs can control the seizure (Agbo et al., 2023). Therefore, several clinically approved AEDs are targeting VGSCs, including the widely used phenytoin, lamotrigine, and carbamazepine. Several drugs that are currently in various phases of clinical trials are also targeting these channels. Much research has been carried out on the use of plant extract and phytochemicals to inhibit VGSCs, which are widely used in various countries for the control of seizures and treated epilepsy.

The USFDA and the European Medicines Agency (EMA) approved cannabidiol (CBD) in the purified form from the cannabis plant against Dravet syndrome and Lennox−Gastaut syndrome and are currently available under the trade name of Epidiolex (Anderson et al., 2021a). The approval and strong anticonvulsive effect of CBD attract the researcher whether other phytocannabinoids also have similar anticonvulsive properties. Therefore, Anderson et al. also evaluated the four different types of lesser studies of phytocannabinoids against different types of epilepsy and found that these compounds show stronger anticonvulsive properties (Anderson et al., 2021a; Anderson et al. 2021b; Anderson et al. 2022). However, the mechanism was unknown. Recently, they hypothesized that as the CBD has Na^+^ channel modulation properties, it is possible that the other phytocannabinoids could modulate the sodium channels. They investigated the mechanism of action of these phytocannabinoids and found that, among five studied compounds, two were sodium channel blockers and had an almost similar mechanism of action as CBD (Milligan et al., 2023). However, further confirmation could be carried out in animal models followed by humans. From these results, we can conclude that the cannabis family has many members of different plant species, and it could contain different types of phytochemicals and metabolites that have similar mechanisms of action and could control the seizure by action on sodium or can treat any other channels that can control the seizures. Saponins are produced by plants and lower animals, as well as bacteria (Singh and Goel, 2016). These play a central role in various diseases, such as hypertension, atherosclerosis, inflammation, cognitive impairments, allergic reactions, and cancer. Singh et al. reported the anticonvulsive effect of saponins extracted from the roots of *Ficus religiosa*. The authors used various epileptic mice models and found that the saponins showed strong anticonvulsive effects in various models. In addition, they concluded that they modulated the GABAergic, Na+, and Ca^2+^. Furthermore, they documented that they only deactivated the VGSCs and VGCCs without affecting the ligand-gated channels (Singh and Goel, 2016). These results are very interesting as they target three different channels and could be used against intractable epilepsy (When an individual seizure does not respond to at least two clinically approved drugs with different mechanisms of action, termed intractable epilepsy). Further research is needed to study this compound in drug-resistant epilepsy models and investigate the safety and efficacy of specific doses. The plant extracts, various isolated phytochemicals, and their mechanism of action on the VGSCs are summarized in Table 3.

Moreover, several studies have been reported on various types of phytochemicals, and medicinal plant extracts *invitro* against epilepsy by modulation of the Na + channels and show promising activities (Lai et al., 2022; Milligan et al., 2022). These plants and compounds could be investigated on the animal models to further evaluate their efficacy, toxicity, safety, and dose determination.

## 8 Modulation of voltage-gated potassium channels

Potassium channels (Kv channels) are the largest family, consisting of twelve sub-families. There is a total of 350 expressed ion channels have been reported in the mammalian brain, of which 145 are voltage-gated channels. Among the voltage-gated channels, 40 are Kv channels, which are further classified into 12 subfamilies (Kv1-Kv12) and represent the largest group of channels (Köhling and Wolfart, 2016). These are activated by the depolarization and deactivated by the repolarization (Köhling and Wolfart, 2016; Ranjan et al., 2019). Kv channels plays a significant role in the homeostasis of the cell’s internal environment by the efflux of potassium (K^+^) ions from the cell and, therefore, plays a role in the control of action potential and hyperpolarization. However, when dysregulation occurs in the normal function of these channels, such as the mutation in the subunits of Kv channels is one of the causes of inherited epilepsy (Nikitin and Vinogradova, 2021). Therefore, these are considered the main targets for the development of drugs against epilepsy. It has been reported that the extract of several plants and phytochemicals has the potential to modulate these receptors to help in the control of seizures (Khan et al., 2020).

4-hydroxybenzoic acid (4-hba) is a benzoic acid derivative and is present in various plants, including *Dendrocalamus asper*, commonly known as bamboo. 4-hba has a lower molecular weight of 138.12 and, therefore, can cross the BBB and cerebrospinal fluid (CSF) (Camusso et al., 2007). In addition, no cytotoxic activities have been reported. Jingli and colleagues investigated the effect of the 4hba on the Kv, and they reported that 4hba enhanced the activity of the potassium channels family Kv1.4 and contributed to the reduction of membrane excitation (Zhang et al., 2018). Therefore, from these results, we can conclude that the 4hba compound could help in controlling the depolarization and excitation of neurons in seizures and, therefore, can be a potent agent against epilepsy in the future. In addition, further investigation can be carried out for the study of other phytochemicals from the same plant and evaluation of their anticonvulsive properties.

*Pseudospondias macrocarpa* is widely used for the treatment of various diseases, especially for neurological disorders in Africa. It has been traditionally reported that, the plant has a sedative effect on the people who can sit or sleep under this plant (Wickens, 1986). The anticonvulsive activity in six various epilepsy animal models has been reported. The authors reported that 30, 100, and 300 mg/kg ethanolic leaves extract significantly increased the seizure onset time and reduced the frequency and duration of the seizures. In addition, the prophylactic use of the extracts significantly improved the survival rate. The authors did not investigate the mechanism of action of the extract; however, from the previous studies on the other plants and phytochemicals from the same groups, they concluded that the promising ability of the plant extract may probably be due to affecting the GABAergic, NMDA, Kv channels, and nitric oxide cGMP pathways (Adongo et al., 2017). However, further studies should be carried out to find out the exact mechanism of action for further use to treat epilepsy and other diseases. Recently, a similar study was reported by Manville et al., 2023. In this study, they used *Salvia rosmarinus*, commonly known as Rosemary extract, against epilepsy. In this article, the authors reported that this plant contains two important compounds, carnosic acid and phenolic diterpene, and both showed promising activities on the selective isoforms of KCNQ (Manville et al., 2023). These compounds could be used as potential antiepileptic agents alone or in combination with other drugs.

The type of drug and dosage varies from person to person based on the types and severity of epilepsy. For example, a combination of drugs will be given to control the seizure. It has also been reported that the phytochemicals in combination with modern drugs have a strong antiepileptic effect as compared to the modern drug alone. Manville and Abbott 2018 conducted a very interesting study in which they used two components, mallotoxin (MTX) and isovaleric acid (IVA), of *Mallotus oppositifolius* leaf extract (Manville and Abbott, 2018). First, the authors evaluated the anticonvulsive effect of these two compounds separately, and they were ineffective. Then they tested the synergistic effect of these two compounds, and interestingly, they showed promising results on the modulation and opening of Kv channels isoform KCNQ2–5, and the activation of these channels is of critical importance in the control of seizure, action potential, and repolarization. After that, they co-administered these two compounds in combination with the modern antiepileptic drug retigabine. From the results, they concluded that the two Phyto flavonoids in combination with the modern molecules were more effective than the two phytocompounds alone (Manville and Abbott, 2018). We can conclude that the medicinal plant extract and phytochemicals have antiepileptic activities alone; however, if we use them in combination with modern drugs, it could further enhance the efficacy of the drug. However, further research could be carried out to determine the effect of the current phytochemicals tested in combination with modern drugs.

Several studies have been reported on the use of plant extract or isolated phytochemicals against various types of epilepsy models. We summarized the recent literature, and Table 3 shows all those plants and phytochemicals that play a significant role in controlling seizures by affecting the Kv channels.

## 9 Antioxidant and anti-inflammatory activity of plant extracts and phytochemicals

We discussed how a specific receptor, channel, or enzymes play a significant role in the development of epilepsy. In addition, we also discussed that epilepsy is basically the imbalance of the excitatory and inhibitory neuronal pathways, in which when excitation occurs then, the inhibitory neurons fail to control the action potential and repolarize the membrane. However, the dysfunction of these molecular entities involved is not only due to mutations in the genes. There are several reasons which directly or indirectly contribute to the development of epilepsy. For example, different types of microorganisms, such as bacteria, viruses, and fungi, can cause epilepsy. When these microorganisms enter the body or specifically the brain, it causes inflammation due to which different types of interleukins or cytokines are released; it also can activate the autoimmune system, which releases antibodies that can attack different types of receptors and contribute to epileptogenesis and hence considered the cause of epilepsy. Vezzani et al., 2016 summarized comprehensive information on the causes and mechanism of infection and inflammation in epilepsy. In addition, some metabolites and other signalling pathways and receptors are directly or indirectly involved. Plants and phytochemicals can also act on these molecules and can help control seizures and treat epilepsy (Vezzani et al., 2016).

The excessive increase of reactive oxygen species (ROS) leads to an increase in levels of Ca^+2^ ions, which play a critical role in the initiation and propagation of seizure, as well as results in the entry of the neurotoxin and inflammatory cells, which are considered mediators of seizure (DeLorenzo et al., 2006). Therefore, the reduction and control of ROS help in the control of seizures. The effect of ferulic acid, a phenolic compound found in many plants, has been evaluated against the PTZ-induced model of epilepsy (Hassanzadeh et al., 2017). The authors administered 70 and 100 mg/kg doses of ferulic acid and reported that both of the doses significantly reduced seizure scores, myoclonic jerks, and cognitive decline. The authors investigated the glutathione (GSH) levels, which are the indicators of ROS, and they found that significant differences were found in the treatment group and PTZ controlled group. They finally concluded that the antiseizure effect of ferulic acid was due to the reduction of GSH content in the brain (Hassanzadeh et al., 2017). In another study, Smilin Bell Aseervatham et al., 2020 evaluated *Passiflora caerulea* L. fruit extract against the PILO-induced model, and it reported that the phytochemicals such as apigenin-6,8-di-C-β-D-glucopyranoside present ameliorate the seizure, cognitive deficit, and oxidative stress (Smilin Bell Aseervatham et al., 2020). Several studies have reported that the phytochemicals target the ROS and reduce the oxidative stress to control the seizure (Table 4).

**TABLE 4:** Effect of plant extracts and isolated phytochemicals from various plants on inflammation, reactive oxygen species, oxidative stress, and other pathways for the control of seizure and treatment of epilepsy.

S. No	Plant	Phytochemical	Plant extract/Isolated phytochemical (s)	Model (s) used	Specie(s) used	Dose (s)	Target(s)/Mechanism of action	References
1	*Zingiber officinale*	Zingerone	Phytochemical	PILO Induced	Male Swiss albino mice	25 and 50 mg/kg	Precluded oxidative stress and inflammation	Rashid et al. (2021)
2	*Passiflora caerulea*	Ginsenoside, naringenin, apigenin-6,8-di-C-β-D-glucopyranoside, chrysoeriol 8-c-glucoside, luteolin-6-C-glucoside	Fruit extract	PILO Induced	Male Swiss albino mice	200 mg/kg	Reduced the oxidative damage	Smilin Bell Aseervatham et al. (2020)
3	*Siparuna guianensis* and *Matricaria chamomilla*	(−)-α-bisabolol	Phytochemical	PTZ Induced	Male Wistar rats	50 and 100 mg/kg	Reduced levels of TNF-α, IL-1β, and MDA oxidative markers	Nazarinia et al. (2023)
4	*Curcuma longa*	Curcumin	Phytochemical	MES Induced	Male Sprague Dawley rats	-	Modulated the MAPK pathway	Drion, et al. (2018)
5	*Cannabis sativa L., Origanum vulgare L*	Trans-caryophyllene	Phytochemical	KA Induced	-	30 and 60 mg/kg	Exerted anti-inflammatory effects by suppression of proinflammatory cytokines, such as TNF-α and IL-1β	Liu et al. (2015)
6	*Maerua angolensis DC*	-	Stem bark extract	PTZ induced	Sprague-Dawley rats	100–1,000 mg/kg	Provided protection against free radicals and the oxidative stress	Benneh et al. (2018)
7	*Scutellaria Baicalensis Georgi*	Baicalein	Phytochemical	PILO induced	Male Sprague-Dawley rats	40 mg/kg	Reduces pro-inflammatory cytokines levels	Qian et al. (2019)
8	Various vegatables	Luteolin	Phytochemical	KA induced	Male Sprague–Dawley rats	10 or 50 mg/kg	Mitigating inflammation, and enhancing Akt activation in the hippocampus	Lin et al. (2016)
9	-	Hispidulin	Phytochemical	KA induced	Sprague-Dawley rats	10 or 50 mg/kg	Suppressed the production of proinflammatory cytokines such as IL-1β, 6, and TNF-α in the hippocampus	Lin et al. (2015)
10	*Piper nigrum*	Piperine	Phytochemical	PILO induced	Sprague Dawley rats	40 mg/kg	Decreased inflammation and oxidative stress	Mao et al. (2017)
11	*Scutellaria baicalensis Georgi*	Baicalin	Phytochemical	PTZ induced	Male Sprague Dawley rats	50 mg/kg	Modulated TLR4/MYD88/Caspase-3 pathway	Yang et al. (2021a)
12	*Anacyclus pyrethrum*	Polyphenols, tannins, coumarins, sterols, triterpenes, alkaloids	Root extract	KA induced	Male Swiss mice	5 g/L	Neuroprotective effect	Manouze et al. (2019)
13	Various plants	Esculetin	Phytochemical	PTZ induced	Male Wistar rats	10 mg/kg	Anti-neuroinflammatory effects	Danis et al. (2023)
14	*Sinomenium acutum*	Sinomenine	Phytochemical	PTZ induced	Male Sprague-Dawley rats	20, 40, and 80 mg/kg	Inhibited NLRP1 inflammasome-mediated inflammatory process	Gao et al. (2018)
15	*Amomum tsaoko*	-	Fruit extract	PTZ induced	Male Swiss albino mice	50, 75, and 100 mg/kg	Suppressed the mRNA expressions of NF-κB, IL-1β, TLR-4, TNF-α, and COX-2	Wang et al. (2021)
16	*Pergularia daemia*	Flavonoids, cardenolides, alkaloids, phenols, saponins, triterpenes, glycosides, carbohydrates, saponin, carbohydrates, glycosides, proteins, cardiac and tannins	Roots extract	PTZ induced	Mice	1.6, 4, 8 and 16 mg/kg	Suppressed oxidative stress, and neuroinflammation	Kandeda et al. (2021a)
17	*Phyllanthus amarus*	Phyllathin	Phytochemical	PTZ induced	Male Swiss albino mice (weight	100 and 200 mg/kg	Downregulated brain mRNA expressions of NF-κB, TNF-α, IL-1β, COX-2, and TLR-4	Tao et al. (2020)
18	*Prunes Alleviates*	Pinoresinol-4-O-β-d-glucopyranoside	Phytochemical	PILO induced	Male Wister rats	50 mg/kg	Alleviation of neuroinflammation	Youssef et al. (2020)
19	*Otostegia limbata*	Phenols and flavonoids	Whole plant extract	PTZ induced	Male Swiss albino mice	100, 200, and 300 mg/kg	Downregulated p-NFκB and TNF-α expression	Amin et al. (2022)
20	*Cnestis ferruginea*	-	Root extract	KA induced	Mice	400 mg/kg	Attenuation of neuro-inflammatory transcription factors	Ojo et al. (2019)
21	*Passiflora incarnata*	Amino acids, and a cyanogenic glycoside gynocardin, nonflavonoid	Flowers extract	PILO induced	Sprague Dawley adult male rats	200 mg/kg	Exerted antioxidant and anti-inflammatory cascade activities	Gad et al. (2022)
22	*Stevia rebaudiana Bertoni*	-	Leaves extract	PTZ induced	Sprague-Dawley rats	200 mg/kg	Downregulated GFAP, IL-6, NF-kB, caspase-3, and p53	El Nashar et al. (2022)
23	*Genipa americana*	Polysaccharides and many more	Leaves extract	PTZ induced	Male Swiss mice	1 or 9 mg/kg	Possess anti-inflammatory and antioxidant activities	Nonato et al. (2024)
24	*Rosa webbiana*	-	Fruits extract	PTZ induced	Male Sprague–Dawley rats	50, 150 and 300 mg/kg	Downregulation of neuro-inflammation, p-TNF-α and p-NF-κB	Firdous et al. (2021)
25	*Echinops spinosus*	Polyacetylene, thiophene, flavone glycoside, alkaloids, and benzothiophene glycoside	Whole plant extract	PTZ induced	Male Wistar rats	250 mg/kg	Exerted neuromodulatory, antioxidant, anti-inflammatory effect	Alkhudhayri et al. (2023)
26	*Nelumbo nucifera Gaertn*	Neferine	Phytochemical	KA induced	Male Sprague-Dawley rats	10 and 50 mg/kg	Inhibiting NLRP3 inflammasome activation and decreasing inflammatory cytokine secretion	Lin et al. (2022)
27	*Glycyrrhiza radix*	Glycyrrhizin	Phytochemical	PTZ induced	Zebrafish	25, 50, and 100 mg/kg	Downregulated level of HMGB1, TLR4, NF-kB, and TNF-α mRNA expression	Paudel et al. (2021)
28	*Albizia adianthifolia*	Apocarotenoids, imidazolyl carboxylic acids, alkaloids, steroids, flavonoids, triterpenoids, elliptosides, fatty acids, saponins, and saponins	Leaves extract	PTZ induced	Male mice *Mus musculus* Swiss	40, 80, or 160 mg/kg	melioration of oxidative stress and neuroinflammation	Nkwingwa et al. (2023)
29	*Rhodiola rosea*	Salidroside	Phytochemical	PTZ induced	Male Wistar rats	50 mg/kg	Activating the Nrf2-ARE signal pathway	Wu et al. (2020)
30	*Ginkgo biloba*	-	Whole plant extract	PILO induced	Male C57BL/6 mice	100 mg/kg	Inhibiting lncRNA-COX2/NF-κB inflammation signalling	Zou et al. (2023)
31	*Psychotria camptopus*	-	Stem bark extract	PTZ induced	Male Wistar rats	40, 80 and 120 mg/kg	Augmentation of antioxidant and neuroprotective defense mechanisms	Fokoua et al. (2021a)
32	Boraginaceae species	Rosmarinic acid	Phytochemical	KA induced	Male Wistar rats	10 or 300 mg/kg	Mitigates oxidative stress	Khamse et al. (2015)
33	*Rosmarinus officinalis*	Homoplantaginin, gallocatechin, 6-hydroxyluteolin-7-glucoside, genkwanin, cirsimaritin, luteolin-3′-glucuronide	Leaves extract	PTZ induced	Male Wistar rats	100 mg/kg	Increased antioxidation	Alrashdi et al. (2023)
34	Various plants	Luteolin	Phytochemical	PTZ induced	Rats	-	inhibition of the TLR4/IκBα/NF-κB pathway	Cheng et al. (2024)
35	*Vanilla planifolia*	Vanillin	Phytochemical	PTZ induced	Male Swiss albino mice	40 mg/kg	Downregulating the HMGB1/RAGE/TLR4/NFκB pathway	Almostafa et al. (2024)
36	Tephrosia species	(−) Pseudosemiglabrin	Phytochemical	PILO induced	BALB/c mice	12.5, 25, or 50 mg/kg	Suppressed TLR-4/NF-κB and the enhancement of the Nrf2/HO-1 and PI3K/Akt pathways	Balaha et al. (2023)
37	*Alchemilla Kiwuensis Engl*	Flavonoids, alkaloids, and tennins	Whole plant extract	PTZ induced	Albinos Wistar rats	40 mg/kg, 80 mg/kg	Modulation of neuroinflammatory pathways	Foutsop et al. (2023)

^a^ For abbreviations, see List of abbreviations.

**FIGURE 6:**
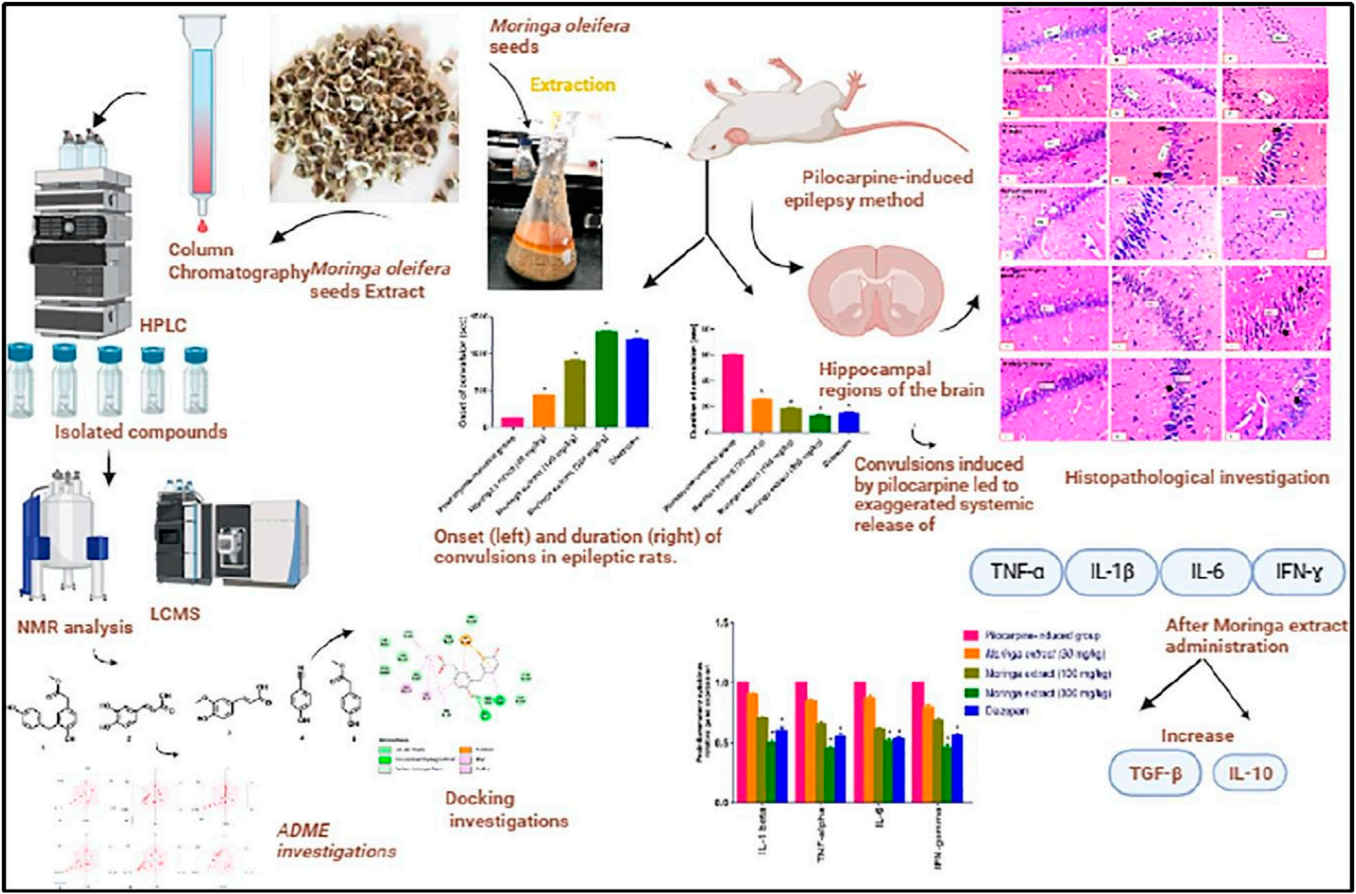
A comprehensive summary of moringa extracts in the rat model of temporal lobe epilepsy (Fayez et al., 2023). Copyright permission@ Elsevier 2023.

Tumor necrosis factor (TNF- α), interferon (IFN- γ), *and interleukins such as IL-6 and IL-1*β *have been reported to cause the failure of the BBB. Similarly, the disparity in pro and anti-inflammatory cytokines further worsens organ damage and is involved in the pathogenesis of epilepsy* (Abdallah et al., 2022)*. IL-6 is a pro-inflammatory cytokine and is considered one of the biomarkers found in the blood and brain of epileptic patients* (Youn et al., 2013)*.* Shaimaa and coworkers conducted a comprehensive study on the use of *Moringa oleifera* seeds extract in pilocarpine-induced epileptic rats with temporal lobe epilepsy (Fayez et al., 2023). The authors used various doses of the extract and reported that the extract extensively modulates the pro and anti-inflammatory cytokines. They further clarify that the extract contained various important phytochemicals that had anticonvulsive effects by suppressing the pro-inflammatory cytokines TNF-α, IL-1β, IL-6, and IFN-ɣ and increasing the levels of anti-inflammatory cytokines TGF-β and IL-10 in the hippocampal tissue of the animal model. The authors also compared the results with the standard drug diazepam treatment group and concluded that the moringa seed extracts possess strong antiepileptic properties and could be used for the treatment of seizures Figure 6 (Fayez et al., 2023). In another study, the authors used *P. daemia* against a PTZ-induced model of temporal lobe epilepsy. They also reported that the extract and reported that the phytochemicals in the extract lead to the suppression of the pro-inflammatory cytokines such as TNF 1β and −6 in the hippocampus and, therefore, lead to a decrease in the latency and duration of seizure and increase the score of seizure. In addition, they further reported that the extract alleviates kainite-induced impairment and finally concluded that the extract possesses strong antiepileptic properties and could be used for the treatment of epilepsy (Kandeda et al., 2021b). *Emblica officinalis* hydrochloric acid extracts also reported that same results in kainic acid induce model of epilepsy. Many studies have reported that plant extracts and phytochemicals could suppress the pro-inflammatory phytochemicals as well as increase the anti-inflammatory phytochemicals (Golechha et al., 2011). Therefore, these plants could be used for the treatment of epilepsy. We summarized some phytochemicals and plant sources that have anti-inflammatory properties and were investigated for the treatment of epilepsy, as shown in Table 4.

In addition, phytochemicals can also target and modulate various signalling pathways, which also play a role in the development and propagation of seizures. Several studies have been reported, including Inhibition of the NF-κB/TLR-4 Pathway (Tao et al., 2020), interaction with CREB-BDNF Pathway (Sharma et al., 2019; Zarneshan et al., 2022), AMPK/PPAR-α (Yang Y. et al., 2021) and AKT/CREB/BDNF (Yu et al., 2019) signalling pathways, and GluR2/ERK I/II pathway inhibition.

## 10 Conclusion and future prospects

Epilepsy is a complex and multifactorial neurological disease in which various receptors, membrane protein channels, enzymes, and pathways are involved in the epileptogenesis and worsening of a disease condition. Although a plethora of drugs are available in the market, but still about 30% of individuals show resistance to the current medication. In addition, these drugs also have some limitations, such as side effects, high price, and availability, especially in low-income countries. Therefore, there is a need for multitargeted approaches that are pocket-friendly, readily available and have no or fewer side effects.

Since immemorial, medicinal plants have been widely used for the treatment of various types of ailments, including neurological disorders, especially epilepsy. There are several advantages of these phytotherapeutic options compared to the synthetic drugs and pharmacological agents as mentioned. Plant extracts and isolated phytochemicals have been widely used in various animal models and have shown promising potential for the control of seizures and the treatment of epilepsy. These plant-based materials can target all the known targets for the currently available AEDs, as shown in Figure 2. In addition, it also targets other important pathways such as inflammatory, oxidative stress, and many others, as shown in section 9, as their inherited properties, such as anti-inflammatory and antioxidants, are mainly responsible for neuroprotective effects as well. Furthermore, the multitargeted potential of these plant-based materials has been observed to be superior to that of synthetic drugs. These can target various types of targets at the same time. For example, the whole plant extract of *Alchemilla kiwuensis* was used against the PTZ-induced epileptic rats model. The authors document that the extract has a significant antiepileptic effect, decreasing GABA-T enzymes, which leads to an increase in the GABA levels in the brain. In addition, it modulates the glutamatergic pathway and possesses anti-neurotic and antioxidant properties. This means that a single plant extract can target four pathways. In the drug resistant epilepsy, which is one of the main focus of the current antiepileptic research in which, two or more drugs with different mechanism of action or targets are used for the treatment. From the current literature, we can conclude that the plant extracts and isolated phytochemicals possess strong antiepileptic properties and could be used for the treatment of epilepsy, especially in lower income countries and patients with drug-resistant epilepsy. WHO also recommends the use of medicinal plants in various healthcare benefits programs in various countries. However, before the use, safety, dosage, exact mechanism of action, and therapeutic efficacy should be properly evaluated.

Until this time, hundreds of plants and their phytochemicals have been evaluated *in vitro* and *in vivo* against epilepsy and showed a potential effect. Unfortunately, only a few phytochemicals enter human clinical trials. Most phytochemicals are used in countries where people have lower purchasing power. Perhaps, in addition to this fact, the lack of investment in basic research, applied research, and folk medicine to understand certain plants also occurs. Similarly, other reasons could be ignorance of side effects, safety, specific dose, cytotoxicity, exact mechanism of action, complete knowledge and structure of the phytochemicals, and many more. Therefore, precise, rigorous, and extensive research could be carried out to investigate all these before entering into clinical trials. In addition, traditional methods for the identification of species are also adequate, and therefore, DNA barcoding could be used for the identification and screening of plant species. In conclusion, medicinal plants could be one of the main treatment options against epilepsy in the near future if we ensure the safety, efficacy, and effective dosage. This treatment strategy will be cost-effective, easily available worldwide, lower toxicity on health cells, and could eliminate the burden of epilepsy.

## Figures and Tables

**Table:** 

**WHO**	World Health Organization
**ILAE**	International League Against Epilepsy
**AEDs**	Antiepileptic Drugs
**NMDAR**	N-methyl-D-aspartate receptor
**GABAR**	gamma-aminobutyric acid receptor
**AMPAR**	α-amino-3-hydroxy-5-methyl-4-isoxazolepropionic acid receptor
**USFDA**	United States food and drug administration
**MES**	maximal electrical shock
**PTZ**	pentylenetetrazol
**L-GAD**	l-glutamate decarboxylase
**TNF**	Tumor necrosis factor
**IFN**	interferon
**IL**	Interleukins
**BBB**	Blood brain barrier
**GSH**	glutathione
**ROS**	reactive oxygen species
**Ca+**	calcium ion
**Na+**	Sodium ion
**K+**	potassium ion
**Kv**	Voltage Gated potassium channel
**VGSCs**	Voltage gated sodium channels
**VGCCs**	Voltage gated calcium channels
**CBD**	cannabidiol
**HPLC/MS**	High performance liquid chromatography mass spectrometry
**EEG**	electroencephalography
**GABA-T**	Gamma-aminobutyric acid transaminase
**PILO**	Pilocarpine
**PIC**	Picrotoxin
**4-AP**	4-aminopyridine
**KA**	Kainic acid
**Nrf2**	Nuclear factor erythroid 2 [NF-E2]–related factor 2
**HMGB1**	High mobility group box 1
**NLRP3**	NOD-LRR- and pyrin domain-containing protein 3
**GFAP**	Glial fibrillary acidic protein
**COX-2**	Cyclooxygenase-2 inhibitors
**MYD88**	Myeloid Differentiation Primary Response Protein 88
**TLR4**	Toll-like receptor 4
**NF-κB**	nuclear factor-kappa B
